# ProgPerm: Progressive permutation for a dynamic representation of the robustness of microbiome discoveries

**DOI:** 10.1186/s12859-021-04061-3

**Published:** 2021-03-17

**Authors:** Liangliang Zhang, Yushu Shi, Kim-Anh Do, Christine B. Peterson, Robert R. Jenq

**Affiliations:** 1 Department of Biostatistics, University of Texas MD Anderson Cancer Center, Houston, TX USA; 2 Department of Statistics, University of Missouri, Columbia, MO USA; 3 Department of Genomic Medicine, University of Texas MD Anderson Cancer Center, Houston, TX USA

**Keywords:** Differential test, Fragility index, Feature selection, Microbiome, Permutation, Robustness

## Abstract

**Background:**

Identification of features is a critical task in microbiome studies that is complicated by the fact that microbial data are high dimensional and heterogeneous. Masked by the complexity of the data, the problem of separating signals (differential features between groups) from noise (features that are not differential between groups) becomes challenging and troublesome. For instance, when performing differential abundance tests, multiple testing adjustments tend to be overconservative, as the probability of a type I error (false positive) increases dramatically with the large numbers of hypotheses. Moreover, the grouping effect of interest can be obscured by heterogeneity. These factors can incorrectly lead to the conclusion that there are no differences in the microbiome compositions.

**Results:**

We translate and represent the problem of identifying differential features, which are differential in two-group comparisons (e.g., treatment versus control), as a dynamic layout of separating the signal from its random background. More specifically, we progressively permute the grouping factor labels of the microbiome samples and perform multiple differential abundance tests in each scenario. We then compare the signal strength of the most differential features from the original data with their performance in permutations, and will observe a visually apparent decreasing trend if these features are true positives identified from the data. Simulations and applications on real data show that the proposed method creates a U-curve when plotting the number of significant features versus the proportion of mixing. The shape of the U-Curve can convey the strength of the overall association between the microbiome and the grouping factor. We also define a fragility index to measure the robustness of the discoveries. Finally, we recommend the identified features by comparing *p*-values in the observed data with *p*-values in the fully mixed data.

**Conclusions:**

We have developed this into a user-friendly and efficient R-shiny tool with visualizations. By default, we use the Wilcoxon rank sum test to compute the *p*-values, since it is a robust nonparametric test. Our proposed method can also utilize *p*-values obtained from other testing methods, such as DESeq. This demonstrates the potential of the progressive permutation method to be extended to new settings.

**Supplementary Information:**

The online version supplementary material available at 10.1186/s12859-021-04061-3.

## Background

With the advent of next-generation sequencing technologies to quantify the composition of human microbiome, there have been drastic increases in the number of microbiome studies and vast improvements in microbiome analysis [1]. In recent decades, a tremendous amount of evidence has strongly suggested that the human microbiota is becoming a crucial key to understanding human health and physiology [2–8]. In practice, identification of microbial biomarkers often requires singling out specific taxa that are differentially abundant between two groups of interest (e.g. treatment vs. control). Differential abundance analysis [9] in this setting, however, is challenging. On the one hand, microbiome data are high dimensional with complex structures. A single sample can produce as many as tens of thousands of distinct sequencing reads. These reads are clustered into operational taxonomic units (OTUs) and mapped to the microbial species according to a reference library. At the same time, the OTUs (which can be considered as the lowest level taxa) are routinely aggregated to higher taxonomic levels (phyla, order, class, family, genus, or species). On the other hand, microbiome data are heterogeneous across subjects that belong to different populations, because microbiome samples interact with different body environment that might be depicted by multiple clinical outcomes. It is highly likely that not all of these host phenotypes are collected and included in the study, but with all the available clinical factors in the current data, we would like to explore and investigate a subset that are most associated with differences in microbiome compositions. Then we would like to identify the corresponding microbiome features that are significantly and robustly associated with these clinical outcomes.

Researchers have adapted classical differential analysis tools developed for RNA sequencing data, such as edgeR [10] and DESeq [11], to microbiome data, as both data types are essentially read count data. Others have proposed methods that account for the compositional nature of microbiome data, including ANCOM [12] and ALDEx2 [13]. Segata et al. [14] developed LEfSe (Linear discriminant analysis Effect Size) to identify differential taxonomic features between groups by using standard tests for statistical significance. When doing multiple tests, the probability of a Type I error (false positive) increases dramatically as high throughput sequencing data is tested [15]. Adjustment methods such as the Benjamini–Hochberg procedure will become over-conservative and incorrectly lead to conclusions that there are no differences in the microbiome, because the threshholds of rejecting the null hypothesis for each microbe becomes extremely small as the number of tests increases [16]. Although these differential testing methods are able to identify the significance of individual microbiomarkers when associating with a single clinical outcome, they do not answer a more general question as to which grouping factors better identify more differences in microbiome communities and deserve further analysis when multiple clinical outcomes are presented in the observed data. Researchers usually use dimension reduction plots (e.g. PCoA or NMDS) at the beginning to explore the overall associations between clinical outcomes and microbiome compositions before any further investigations. But the expected clustering effect may or may not be observed depending on the degree of heterogeneity across samples and populations, which could lead to the false conclusion that the microbiome is not associated with a clinical factor. Therefore, a systematic tool is needed to explore both the overall and the individual associations, and to provide measures on the robustness of the discoveries and the reliability of the results.

We propose a novel method named progressive permutation. The method progressively permutes the grouping factor labels of microbiome samples and performs differential testing (such as a Wilcoxon rank-sum test or a Kruskal–Wallis test) on the permuted data in each scenario. We then compare the signal strength ($-\,\log _{10} p$-values) of top hits from the observed data with their testing performance in permuted data sets. We can observe an apparent decreasing trend of the signal strength from the no permutation scenario to the full permutation scenario, if these top hits are true positives identified from the data. As the fragility index is a measure of the robustness of the results of a clinical trial [17, 18], we propose a similar concept in our progressive permutation to measure the minimum number of permutation steps that would change the variable’s significance to nonsigificance. We also extend these concepts to a continuous outcome using correlation tests (such as Kendall’s tau or Spearman Rank Correlation tests). We have developed this method into a user-friendly and efficient RShiny tool with visualizations, so that the method becomes easy to apply, the results are easy to understand and the process of analyzing is well organized. Hawinkel et al. [19] proposed a permutation filtering method to measure the taxa importance by the filtering loss of exclusion of the taxa. The method randomly permutes the labels of taxa and evaluates the proportion of total variance loss. Our method permutes the sample labels to regroup them and evaluate the robustness of group differences. We validate our method with simulations and applications in real data. We conclude that the proposed method can not only compare the overall association between the microbiome and multiple grouping factors (that might be obscured by heterogeneity), but also single out the robust individual hits. It achieves the former by measuring the changing trend of the number of significant hits across permutation scenarios and ranking the fragility index of the discovered microbes. It achieves the latter by comparing the *p*-values of the observed data (signals) with *p*-values of the fully mixed data (noise). To finalize the results, the RShiny tool lists the discoveries, their effect sizes and individual abundances.

The paper is organized as follows. In “Methods” section, we include a detailed description of the proposed method. In “Simulations” section, we run simulations, and use the U-Curve and fragility index to measure overall associations with grouping factors and the robustness of microbiome discoveries. In “Application” section, we apply the method to real data to test overall associations and identify robust hits. In “Analytical property” section, we show the analytical properties of the proposed method in a simple setup. We conclude with a discussion in “Discussion” section.

## Methods

Suppose that we collect *N* samples and obtain *p* microbiome taxa. We denote the microbial features as ${{\varvec{X}}}=({{\varvec{x}}}_1,\ldots , {{\varvec{x}}}_p)$, where each ${{\varvec{x}}}_i$ is an *N*-dimensional vector. We aim to identify which variables are differential by the grouping factor of interest with two groups ${{\varvec{g}}}=({{\varvec{g}}}^1,{{\varvec{g}}}^2)$ (e.g. ${{\varvec{g}}}^1$ denotes the treatment group while ${{\varvec{g}}}^2$ denotes the control group). We denote the grouping labels in group 1 as $g^1_i=1, i= \{1,\ldots , n_1\}$ and group 2 as $g^2_i=2, i= \{1,\ldots , n_2\}$, where $n_1+n_2=N$. The hypothesis test performed on each variable is denoted as $H_j, j=\{1,\ldots , p\}$. The corresponding *p*-value is denoted as $p_j, j=\{1,\ldots , p\}$.

We use $k=\{0, 1,\ldots , K\}$ to describe progressive permutation scenarios. $k=0$ describes the observed data without any permutation. $K={\mathrm {min}}(n_1, n_2)$ is the maximal permutation scenario. The permutation scenario *k* is constructed as follows. Each time, we start from the original grouping labels ${{\varvec{g}}}=({{\varvec{g}}}^1,{{\varvec{g}}}^2)$. We randomly draw *k* samples from group 1 (sample labels $\{i_1^1,\ldots ,i_k^1\}\subseteq \{1,\ldots , n_1\}$) and *k* samples from group 2 (sample labels $\{i_1^2,\ldots , i_k^2\}\subseteq \{1,\ldots , n_2\}$), and then exchange their grouping labels, meaning that $g^1_{i}=2, i=\{i_1^1,\ldots ,i_k^1\}$ and $g^2_{i}=1, i=\{i_1^2,\ldots , i_k^2\}$. In the *k*-th permutation scenario, we have $$\left( {\begin{array}{c}n_1\\ k\end{array}}\right) \left( {\begin{array}{c}n_2\\ k\end{array}}\right)$$ choices. The number of choices $$\left( {\begin{array}{c}n_1\\ k\end{array}}\right) \left( {\begin{array}{c}n_2\\ k\end{array}}\right)$$ approaches its maximum, when *k* equals the closest integer greater than $\frac{n_1n_2-1}{n_1+n_2+2}$. We call it as the full permutation scenario with $K_f=\lceil \frac{n_1n_2-1}{n_1+n_2+2}\rceil$. If $n_1=n_2=n$, then $K_f=\lceil \frac{n-1}{2}\rceil$. Adding up the choices of all the scenarios, we get the following equation

1 $$\begin{aligned} \sum _{k=0}^{K} \left( {\begin{array}{c}n_1\\ k\end{array}}\right) \left( {\begin{array}{c}n_2\\ k\end{array}}\right) =\left( {\begin{array}{c}N\\ K\end{array}}\right) . \end{aligned}$$

The above equation can be derived from Vandermonde’s convolution identity for binomial coefficients. The details are shown in Additional file 1: Sect. S1. The left side lists all the progressive permutation scenarios which are disjoint meaning that grouping labels are distinct between scenarios. The right side lists all possible combinations when you group *N* samples into two subgroups with $n_1$ and $n_2$ samples respectively. With the increase of *k*, the two groups are mixing more with each other. In other words, among all the grouping assignments at random, the permuted assignments more similar to the original data (the observed grouping factor) would differentiate the two groups more than the less similar ones, if the microbiome variables were strongly associated with the observed grouping factor.

**Fig. 1:**
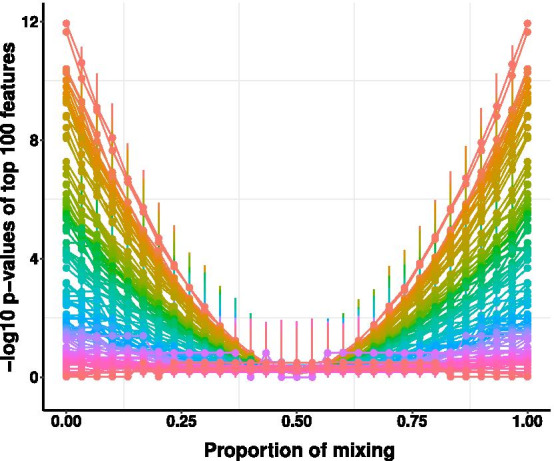
Trace plot of $-\,\log _{10} p$-values changing with the proportion of mixing. x-axis denotes the proportion of mixing. y-axis denotes the $-\,\log _{10} p$-values of the 100 features. Each curve denotes the trace of $-\,\log _{10} p$-value of an individual microbiome feature. The scale of rainbow colors shows the contrast of curves that $-\,\log _{10} p$-values of more significant variables will be higher than those of less significant ones. The vertical bars describe the 95% quantile confidence intervals of the $-\,\log _{10} p$-values across permutation scenarios

Next we introduce how to perform differential tests and utilize the testing results from all the progressive permutation scenarios. In both the permuted and unpermuted data, we perform differential testing of each feature, and obtain the corresponding $-\,\log _{10}p$-values. By default, we use the Wilcoxon rank sum test to compute the *p*-values, since it is a robust nonparametric test. Our proposed method can also utilize *p*-values obtained from other testing methods, such as DESeq [20].

Each permutation scenario consists of multiple combination choices, implemented as follows. For each permutation scenario *k* ($k\ge 1$), we start from a random seed and perform a subset of $$\nu =N\left( \log \left( {\begin{array}{c}n_1\\ k\end{array}}\right) +\log \left( {\begin{array}{c}n_2\\ k\end{array}}\right) \right)$$ (rounded to the nearst integer) draws out of a total of $$\left( {\begin{array}{c}n_1\\ k\end{array}}\right) \left( {\begin{array}{c}n_2\\ k\end{array}}\right)$$ draws. For each draw in every scenario *k*, we perform *p* independent tests to differentiate each microbiome features between the two groups and calculate all the *p*-values. Therefore, for each variable *j* ($j=1,\ldots ,p$), we obtain $\nu$ samples of *p*-values $p_{j}(k)$. We summarize the distribution of these samples by their medians $p^m_j(k)$ and 2.5–97.5% quantile intervals. To visualize these *p*-values in an organized manner, we rank the *p*-values (defined as $p_{j}(0)$) of all the variables in the observed data, and then plot their $-\,\log _{10}$ median *p*-values with the same order across permutation scenarios. Please note that the observed data can be considered as one draw ($\nu =1$), so the median *p*-value $p^m_{j}(0)$ is equivalent to the *p*-value $p_{j}(0)$. As illustrated in Fig. 1, we presented the traces of $-\,\log _{10} p$-values for an example data set with 100 microbiome features. In general, the paralleled traces of $-\,\log _{10}$ median *p*-values of more significant variables will be higher than those of less significant ones. With the increase of mixing, the significant *p*-values gradually become nonsignificant, indicating that the signal is weaker and the noise is stronger. As there would be almost no signal if the data were fully mixed, more *p*-values are close to 1 at the full permutation scenario $k=K_f$. We describe the computational scaling of the progression permutation approach in Additional file 1: Sect. S2.

For microbiome data, the number of taxa *p* is usually a larger number. It is not easy to display and compare a large number of traces. So we summarize individual *p*-values into a single quantity, the number of significant taxa. We can obtain the number of significant taxa as ${\mathrm {nsig}}(k)=\sum _{j=1}^p I_{p^m_j(k)\le \alpha }$, where $\alpha$ is the prespecified significance level (default value is 0.05). We expect to see the lowest ${\mathrm {nsig}}(k)$ in the full permutation scenario $K_f$, because more *p*-values become close to 1 here. The number of significant features ${\mathrm {nsig}}(k)$ decreases with the proportion of mixing *k*/*K*, when $k\le K_f$. ${\mathrm {nsig}}(k)$ increases with the proportion of mixing *k*/*K*, when $k\ge K_f$. If the two groups have balanced sample sizes (i.e. $n_1=n_2$), we will visualize a symmetric U-shape curve if we plot the number of significant features with the proportion of mixing *k*/*K*. The shape of U-Curve measures the signal strength how differential the microbiome compositions are between two groups. We potentially can use the U-Curve as a global measure to depict the overall association between microbiome compositions and different clinical outcomes.

**Fig. 2:**
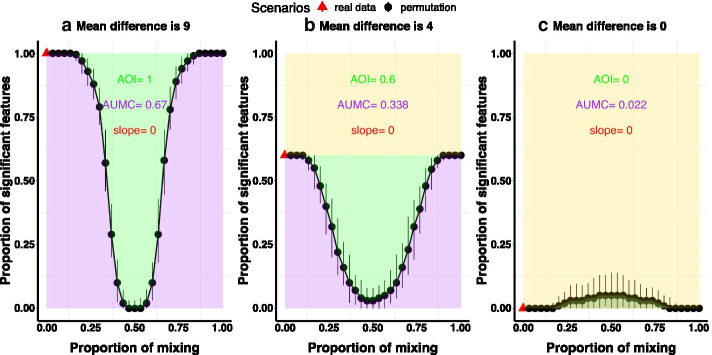
An illustration plot of the U-Curve of proportion of significant features versus proportion of mixing. x-axis describes the proportion of mixing the two groups of data. y-axis describes the proportion of significant features. The red triangle describes the observed data. The black dots describe the permuted data. The vertical bars describe the 95% quantile confidence intervals

To allow the U-Curve comparable across various data sets with different number of microbiome features, we scale the number of significant features ${\mathrm {nsig}}(k)$ by total number of features considered *p*, which is named as the proportion of significant features ${\mathrm {nsig}}(k)/p$ (ranges from 0 to 1). As illustrated in Fig. 2, we define the area of interest (AOI) as the rectangular region covering the curve (green plus purple), which actually measures the proportion of significant features ${\mathrm {nsig}}(k)/p$. In order to describe the shape of the U-Curve, we define area under the mixing curve (AUMC) and the decreasing slope of the initial point depicting the observed data. The AUMC measures the purple area in Fig. 2, which can be calculated numerically. The slope of the initial point is calculated as the slope of the line connecting the first two points. Bigger AUMC means that the number of significant features varies more considerably from the observed data ($k=0$) to the fully mixed data ($k=K_f$), which indicates the higher association between clinical outcomes and microbiome compositions in the observed data. For two clinical outcomes giving equal AOIs, if one outcome provides smaller slope (meaning the signal is stable at the beginning) and bigger AUMC, we will conclude that the overall association between this outcome and the microbiome features were higher.

The fragility index was originally defined as a measure of the robustness of the results of a clinical trial [17, 18]. We introduce a similar concept to measure how fast the signals break down as the mixing increases. We introduce and define the fragility index of *j*th variable of each draw at permutation scenario *k* as ${\mathrm {FI}}_j=\min _k \left( p^m_j(k)>\alpha \right)$, where $p^m_j(k)$ is the median *p*-value obtained above in each scenario *k*. In other words, the fragility index of a variable is the minimum number of permutation steps that would change the variable’s significance into nonsignificance. So the fragility index is smaller than full permutation scenario $K_f$, where all *p*-values are not significant. Therefore, we can obtain the scaled fragility index as ${\mathrm {sFI}}_j={\mathrm {FI}}_j/K_f$. The larger the fragility index is, the more stable the identified taxa are. Therefore, within the same data set, we can rank the importance of the taxa by their fragility indices. For two clinical outcomes, if one outcome is more associated with microbiome features, this outcome will provide higher average fragility indices.

If we roll back the wheel of our proposed method (i.e. Eq. 1), we will find an analogy to scientific research that permuting grouping labels actually lists all the possible arrangements of observations from the same random phenomenon. However, in a single study, researchers observe merely one arrangement, and expect this occasional arrangement among all the others could convey the signal that the two groups are differential. We propose progressive permutation to recover the missing arrangements. We assume that the observed data are differential between the two groups. Then the method generates all the other disjointed arrangements in a systematic manner with fixed sample sizes so that the signal progressively diminishes from the no-permutation scenario (the observed data) to the full-permutation scenario. In other words, if the grouping factor is associated with the microbial difference between the two groups, the observed data defining the signals will be readily able to distinguish from the fully mixed data which characterizes the noise. Therefore, we achieve the identification of robust variables by judging that the significant *p*-values obtained from the observed data lie outside of the 95% confidence intervals of the fully mixed data.

## Simulations

In this section, we first generate two types of simulations to show the performance of our method. First, we change the group mean, variance, correlation and number of significant variables to simulate data with different levels of signals. Second, we control the number of significant variables and simulate three data sets with different levels of heterogeneity. Then we compare the performance of our progressive permutation method on these data.

**Table 1:** Comparison on progressive permutation results produced by multiple simulated data sets with different simulation parameters, including correlation $\rho$, number of significant variables (nsv), group mean difference ($m_1-m_2$), and dispersion $\kappa$

$\rho$	nsv	$m_1-m_2$	$\kappa$	AOI	AUMC	${\mathrm {slope}}_0$	${\mathrm {slope}}_1$	Fragility	${\mathrm {select}}_0$	${\mathrm {select}}_1$
0.5	30	9	24	0.30	0.23	0	− 0.54	6.64	30	30
		9	1	0.30	0.20	0	− 0.54	5.74	30	30
		4	24	0.30	0.18	0	− 0.52	5.96	30	30
		4	1	− 0.05	− 0.05	0.45	0	1.44	5	3
		0	24	0.00	− 0.02	0	0.1	0.44	0	0
		0	1	0.00	− 0.02	0	0.1	0.46	0	0
	90	9	24	1.00	0.67	0	− 2	11.02	100	100
		9	1	1.00	0.58	0	− 1.98	10.14	100	100
		4	24	0.89	0.44	− 0.6	− 1.74	8.84	89	84
		4	1	− 0.08	− 0.08	1.5	− 0.08	2.06	8	3
		0	24	0.00	− 0.02	0	0.1	0.34	0	0
		0	1	0.00	− 0.02	0	0.1	0.5	0	0
0.8	30	9	24	0.30	0.23	0	− 0.54	6.58	30	30
		9	1	0.30	0.20	0	− 0.54	5.8	30	30
		4	24	0.30	0.18	0	− 0.52	5.14	30	30
		4	1	0.04	0.04	− 0.45	0.02	1.2	4	0
		0	24	0.00	− 0.02	0	0.1	0.66	0	0
		0	1	0.00	− 0.02	0	0.1	0.3	0	0
	90	9	24	1.00	0.67	0	− 2	11.12	100	100
		9	1	1.00	0.58	0	− 1.98	10.1	100	100
		4	24	0.97	0.45	− 2.1	− 1.9	8.82	97	87
		4	1	− 0.07	− 0.08	2.25	− 0.04	2.06	7	0
		0	24	0.00	− 0.02	0	0.08	0.54	0	0
		0	1	0.00	− 0.02	0	0.1	0.2	0	0

AOI is short for area of interest. AUMC is short for area under the mixing curve. “${\mathrm {slope}}_0$” denotes the slope of the first point in the U-Curve of number of significant features (the slope of the line connecting the first two points). “${\mathrm {slope}}_1$” denotes the average value of the slope of the first 15 points ($K_f=15$) in the U-Curve of number of significant features (the slope of the line connecting the point with its next neighbor). “fragility” denotes the average value of the fragility index of the first 50 microbiome features. “${\mathrm {select}}_0$” denotes the number of *p*-values that are less than 0.05 given by the testing results on the observed data. “${\mathrm {select}}_1$” denotes the number of significant features identified by the proposed method

We follow the same simulation setup used by [21]. We simulate the OTU counts as random samples drawn from a negative binomial distribution ${\mathcal {F}}(m, \kappa )$, where $\kappa$ is called the dispersion parameter, as the variance is $m+\frac{m^2}{\kappa }$. To simulate the dependence between OTUs, we use the Gaussian copula [22] to combine the correlation structure ${{\varvec{R}}}$ with the negative binomial distributions. Here are the simulation steps. First, we draw Gaussian samples of ${{\varvec{Z}}}\sim {\mathcal {N}}(0,{{\varvec{R}}})$. Second, we obtain the negative binomial samples ${{\varvec{X}}}_j={\mathcal {F}}^{-1}(\Phi ({{\varvec{Z}}}_j)), j =1,\ldots , p$. $\Phi (\cdot )$ denotes the Gaussian cumulative distribution function. Third, we obtain the compositions by dividing each element $X_{ij}$ by a constant greater than the sum of each rows.

To gain a sense of how the shape of the U-Curve depicts the strength and robustness of signals, we construct multiple data sets, changing the simulation parameters and performing progressive permutation on each data set. Let $x_{ij}^1 \sim {\mathcal {F}}(m_j^1, \kappa _j^1)$ denote the simulated data from Group 1. Let $x_{ij}^2 \sim {\mathcal {F}}(m_j^2, \kappa _j^2)$ denote the simulated data from Group 2. The two groups have the same sample size $n_1=n_2=30$ and the same correlation structure as $R_{ij}=\rho ^{i-j}$. We simulate the grouping factor of interest *y* as $[1,\ldots , 1, 2,\ldots , 2]$. Suppose both group consist of 100 variables. Let “nsv” denote the number of differential variables whose distribution means are $m_j^1$ or $m_j^2$, the means of all the other variables is set as 1. As shown in Table 1, we set the means of Group 1 as $\{10,10,10\}$ and the means of Group 2 as $\{1,6,10\}$, so the mean differences between the two groups are $\{9,4,0\}$. For instance, a data set is generated with $m_1-m_2=9$ and nsv=30, meaning that 30% of the 100 variables have strong differences ($m_j^1=10$ vs. $m_j^2=1$, where $j= 1,\ldots , 30$) between the two groups, while all the other 70 variables are not differential (mean difference is 0) between the two groups. We summarize the following observations based on the above simulations. AOI in general increases with the proportion of significant features in the simulated data. As the variance increases when $\kappa$ becomes smaller, the differential effect between the two groups shrinks with $\kappa$. So the AUMC and average fragility of the first 50 features become smaller. The differential effect increases with the two mean differences between the two groups. So the corresponding AUMC and average fragility of the first 50 features become smaller when mean differences are smaller. As shown in Fig. 2, the shape of U-Curve becomes flatter when two groups are less differential. Therefore, the more a grouping factor differentiates the features, the bigger AOI, AUMC and fragility index will be obtained. In particular, when the mean difference between the two groups is close to 0, the AOI and AUMC are almost zero, indicating that the U-Curve of number of significant features is flat when there are no differential signals. Additionally, correlations between microbiome features do not affect the values of the AOI and AUMC. The significant features identified by the proposed method is a subset of features whose *p*-values are less than 0.05 in the observed data.

However, the behavior of steepness of the U-Curve is not clear in the previous simulations. In the following simulations, we control the data to produce the same AOI, but with different slopes. In other words, the number of identified features are the same, but actually the robustness of these features are different. Rather than just consider the significance depicted by *p*-values, we can further consider robustness to evaluate the feature-outcome associations using the U-Curve and fragility index from progressive permutation. We will show that some unknown heterogeneity might be one reason affecting the robustness of the features that are identified as differential. We generate three simulation data sets, which are denoted as SimData 1, SimData 2 and SimData 3. They have the same sample size $n_1=n_2=30$ and same number of variables $p=100$. The 60 samples differ substantially between Group 1 (30 samples) and Group 2 (30 samples). We denote data of Group 1 as $D_1$ and data of Group 2 as $D_2$. For the 100 variables, we define the proportion of significant features to be 0.6, which implies that 60 variables are significant. To construct heterogeneity, we create the second source of difference by splitting Group 1 into two subgroups of samples, which are denoted as $D_{11}$ and $D_{12}$. In the same way, we split Group 2 into two subgroups of samples, which are denoted as $D_{21}$ and $D_{22}$. The grouping factor of interest *y* is $[1,\ldots , 1, 2,\ldots , 2]$.

**Fig. 3:**
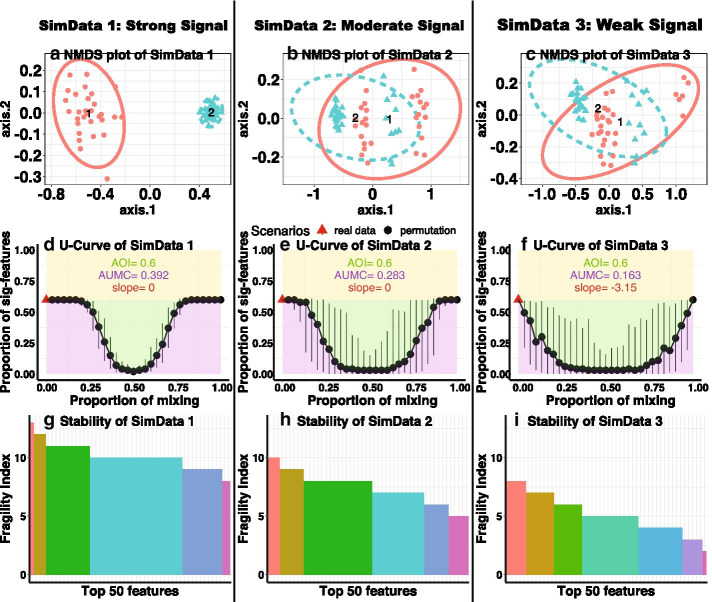
Result comparisons of three simulated data sets with different levels of heterogeneity. The first row (**a**–**c**) shows the NMDS plot using the Bray-Curtis distance. The second row (**d**–**f**) shows the U-Curve of proportion of significant features. AOI is short for area of interest, which denotes the proportion of significant features out of all the features (area of green plus purple). AUMC is short for area under the mixing curve, which denotes the area under the U-Curve (area of purple). Slope denotes the slope of the red triangle. The red triangle denotes the real data. The third row (**g**–**i**) shows the fragility index. The height of each bar represents the Fragility Index value for a given feature. Each color denotes features that have the same levels of fragility. To save space, the legend listing the names of the 50 features are omitted

We describe the data generation as follows. We use $(m)_c$ to denote a sequence containing *c* number of *m*. ${\mathrm {RN}}(\mu _0, \sigma _0)$ describes the random number drawn from normal distribution with mean $\mu _0$ and variance $\sigma _0$. We define the correlation structure as $R_{ij}=\rho ^{i-j}$. $\rho$ is set up as 0.5. Zero-Inflation is one of the main characteristics of microbiome data. Note that $\mu$ controls the magnitude of each variable and number of zeros in each sample. The distribution of zeros across samples and variables of SimData 1, SimData 2 and SimData 3 is comparable to the distribution of zeros in real Data, please see the histograms in Additional file 1: Sect. S3.

- *SimData 1*: $D_{11}$ contains 8 samples. The mean is $[(6)_{30}, (4)_{30}, (1)_{40}]$. The dispersion parameter $\kappa$ is 2. $D_{12}$ contains 22 samples. The mean is $[(4)_{30}, (6)_{30}, (1)_{40}]$. The dispersion parameter $\kappa$ is 36. $D_2$ contains 30 samples. The mean is $[(15)_{30}, (0.5)_{30}, (1)_{40}]$. The dispersion parameter $\kappa$ is 36.
- *SimData 2*: $D_{11}$ contains 16 samples. The mean is $[(8)_{30}, (2)_{30}, (1)_{40}]$. The dispersion parameter $\kappa$ is 25. $D_{12}$ contains 14 samples. The mean is $[(2)_{30}, (8)_{30}, (1)_{40}]$. The dispersion parameter $\kappa$ is 24. $D_{21}$ contains 20 samples. The mean is $[(15)_{30}, (0.5)_{30}, (1)_{40}]$. The dispersion parameter $\kappa$ is 26. $D_{22}$ contains 10 samples. The mean is $[(m_1)_{60},(m_2)_{40}]$, where $m_1= {\mathrm {RN}}(5, 1.2)$ and $m_2={\mathrm {RN}}(1,0.1)$. The dispersion parameter $\kappa$ is 24.
- *SimData 3*: $D_{11}$ contains 24 samples. The mean is $[(8)_{30}, (2)_{30}, (1)_{40}]$. The dispersion parameter $\kappa$ is 14. $D_{12}$ contains 6 samples. The mean is $[(1)_{30}, (10)_{30}, (1)_{40}]$. The dispersion parameter $\kappa$ is 14. $D_{21}$ contains 20 samples. The mean is $[(15)_{30}, (0.5)_{30}, (1)_{40}]$. The dispersion parameter $\kappa$ is 14. $D_{22}$ contains 10 samples. The mean is $[(m_1)_{60},(m_2)_{40}]$, where $m_1={\mathrm {RN}}(5, 1.6)$ and $m_1={\mathrm {RN}}(1, 0.3)$. The dispersion parameter $\kappa$ is 12.

Based on the above setup, we expect to see there are more and more levels of heterogeneity by constructing subgroups from SimData 1 to SimData 2 to SimData 3. As a result, the associations between the microbiome features and the grouping factor of interest is weaker and weaker because the proportion of differential samples between Group 1 and Group 2 is lower and lower. Traditionally, non-metric multidimensional scaling (NMDS) is used to collapse information from multiple dimensional features into just a few, so that clustering effect will be visualized and interpreted when we link them with a grouping factor of interest [23]. However, in the dimension reduction plots, the expected clustering effect can not be witnessed, because this main differential effect is mixed with heterogeneity. As shown in Fig. 3, only the NMDS plot of SimData 1 shows us the clear cluster separations between Group 1 and Group 2. But both the NMDS plot of SimData 2 and the NMDS plot of SimData 3 show overlaps of Group 1 and Group 2 similarly. Therefore, NMDS plots could not distinguish the strength of the overall association between microbiome compositions and the grouping factor of interest. Besides, we can not visualize differences in heterogeneity between SimData 2 and SimData 3.

When testing the relationship between an explanatory variable and an outcome, the variable’s effect might be modified by other variables and distorted by potential systematic bias, confounding or effect modification. The U-Curve and fragility index plots provides us with a measure of all these disturbances mixed with the main signals in the collected data. The U-Curve provides a dynamic depiction of how our method progressively singles out signals from randomized trials. In each plot, the number of significant features decreases from observed data to full permutation scenario. The shape becomes steeper when the associations are less stable (with more disturbances). We use AUMC (area under the mixing curve) to quantify the shape of the U-Curve. AUMCs in Fig. 3d–f are 0.392, 0.283 and 0.163, which ranks the decreasing order of robustness of the association between microbiome compositions and the grouping factor. The average fragility index of the top 50 features are 10.12 for SimData 1, 7.44 for SimData 2, and 5.24 for SimData 3. Since the full permutation scenario $K_f=15$, the average scaled fragility indices are 0.675 for SimData 1, 0.496 for SimData 2 and 0.349 for SimData 3.

Please note that, when generating the U-Curve plots (d–f in Fig. 3), the black dots describe the median value. The black bars describe the $2.5\%$ and $97.5\%$ quantile intervals. We follow the same setup in all the subsequent figures.

**Table 2:** Comparisons of identification performance among different methods.

Data	nsv	Method	FP	FN	Sensitivity	Specificity	Accuracy	RC
Set 1	70	WilPerm	1	3	0.96	0.97	0.96	0.80
		DESPerm	3.5	33.6	0.52	0.88	0.63	0.50
		DESeq	2	0	1	0.93	0.98	0.55
		LEfSe	19	2	0.97	0.37	0.79	0.16
		Logistic	1	4	0.94	0.97	0.95	0.58
	30	WilPerm	3	6.3	0.79	0.96	0.91	0.004
		DESPerm	2.1	12.8	0.57	0.97	0.85	0.094
		DESeq	4	0	1	0.94	0.96	0.17
		LEfSe	10	5	0.83	0.86	0.85	0.24
		Logistic	1	5	0.83	0.99	0.94	0.07
Set 2	70	WilPerm	6.3	0	1	0.79	0.94	0.02
		DESPerm	30	6.3	0.91	0	0.64	0.79
		DESeq	30	9	0.87	0	0.61	0.81
		LEfSe	30	4	0.94	0	0.66	− 0.04
		Logistic	5	40	0.43	0.83	0.55	− 0.88
	30	WilPerm	7	0	1	0.9	0.93	0.35
		DESPerm	19	0	1	0.73	0.81	0.80
		DESeq	3	7	0.77	0.96	0.90	0.80
		LEfSe	70	5	0.83	0	0.25	0.08
		Logistic	7	5	0.83	0.9	0.88	− 0.79

“WilPerm” stands for progressive permutation equipped with Wilcoxon tests. “DESPerm” stands for progressive permutation equipped with DESeq method. FP denotes number of false positives. FN denotes number of false negatives. Sensitivity measures the proportion of positives that are correctly identified. Specificity measures the proportion of negatives that are correctly identified. Accuracy measures the proportion of true positives and true negatives. RC denotes the rank correlation (Spearman’s $\rho$) between the true and estimated ranks of the features. Set 1 denotes the simulation data that varies the zero inflation parameter for each variables. Set 2 denotes the simulation data that varies the mean difference parameter for each variables

In applying our proposed progressive permutation method, we consider *p*-values obtained using the Wilcoxon test and DESeq. Specifically, for DESeq, we rely on the DESeq2 package in R [11], with multiplicity-adjusted *p*-values used to determine hits. We consider features to be significant if their $-\,\log _{10}p$-values in the unpermuted scenario lie outside the 95% quantile intervals of those in the full permutation scenario. We apply these two permutation methods, as well as the standard versions of the DESeq, LEfSe and logistic regression methods, to the simulated data.

We now describe our data generation procedure. Microbiome data are typically overdispersed and zero-inflated. Since the negative binomial distribution can not capture excess zero values, we use another generation mechanism “sparseDOSSA” (https://huttenhower.sph.harvard.edu/sparsedossa/) to allow zero-inflation in the simulated data. In our simulation, we consider a setting with 60 samples (30 samples in each group) and 100 variables. The simulated abundance of each microbial variable is jointly controlled by three parameters: the proportion of zero inflation, mean, and variance.

To study the impact of excessive zero values on the performance of the testing methods, we keep the mean difference (between two groups) and variance to be the same for each variable. We fit the data generation model to a subset of the DeFilippo data (see “Application” section) and obtain the zero inflation parameters for 100 variables. Then we rank the estimated values of the zero inflation parameter from lowest to highest, so that the mean abundances of the simulated data have a decreasing order from the first to the last variable. Let ${\mathrm {nsv}}$ denote the number of variables that are truly differential. We set the mean parameter of the first ${\mathrm {nsv}}$ true variables as 3 in Group 1, and as 0 in Group 2. The variance parameter is set as 1 for all variables. With this setup (named as Set 1), the variables with a smaller zero inflation parameter should be more differential than the ones with a bigger zero inflation parameter.

We also consider an alternative setup (named as Set 2), to observe how the testing methods perform with changes to the mean differences. In this setting, we fix the proportion of zero-inflation (set as 0.1) and variance (set as 0.2) to be the same for each variable. We make a decreasing order of the mean differences from the first to the last variable. With this setup, the variables with bigger mean differences should be more differential than the ones with smaller mean differences. We also design comparisons between data sets with dense signal and sparse signal. For the data with dense signal, the first 70 variables (${\mathrm {nsv}}=70$) are simulated to be differential. For the data with sparse signal, the first 30 variables (${\mathrm {nsv}}=30$) are simulated to be differential.

We report the mean values for all the performance measures in Table 2. FP stands for false positives, where the method identifies a feature that is truly non-differential. FN stands for false negatives, where the method does not identify a feature that is truly differential. RC denotes the rank correlation (Spearman’s $\rho$) between the true and estimated ranks of the features. When comparing the two permutation methods, the version based on the Wilcoxon test achieves higher accuracy than the version based on DESeq. This is likely because the Wilcoxon test is a non-parametric test, while DESeq is a parametric test that requires distributional assumptions. As shown in the U-Curve plots of the number of significant features (Additional file 1: Figs. S4, S6, S8 and S10), the number of significant features does not approach zero in the full permutation scenario when the data are highly zero inflated. This result suggests that the DESeq method incorrectly identifies noise as signal when the data do not follow the assumed distribution. In brief, permutation with the Wilcoxon test is more flexible in dealing with data with unknown complex distributions.

When comparing the proposed permutation method with standard versions of DESeq and LEfSe, we notice that DESeq has high specificity in Setting 1, but an increased rate of false positives in Setting 2, due to violations of its parametric assumptions. Compared with the other methods, LEfSe is too generous, with a high number of false positives. LEfSe uses both *p*-values and effect size to determine hits; however, it does not adjust the *p*-values for multiplicity, and our results suggest that the default threshold on the effect sizes may be overly generous. We also notice that logistic regression has high false negatives in Setting 2. Logistic regression treats the binary outcome as the response variable and the microbial features as the independent variable. This model assumes a linear relationship between the logit of the response variable and the predictors, and may not perform well when this assumption is violated.

**Fig. 4:**
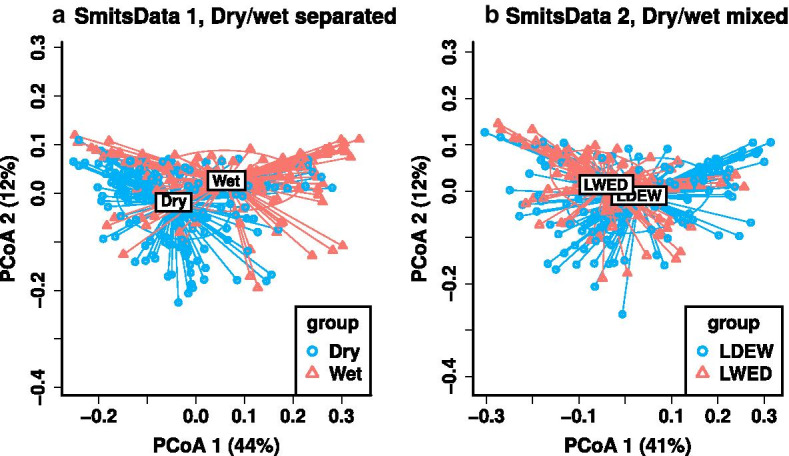
Comparisons of grouping effect in SmitsData 1 (**a**) versus SmitsData 2 (**b**) using PCoA plot. The length of straight line denotes the distance of each individual point to the centroid. The centroid of each group is labeled. The ellipse denotes a 1 standard deviation to the centroid. In plot A, the blue circle denotes the PCoA score of Dry groups, while the red triangle denotes the PCoA score of Wet groups. In plot B, the blue circle denotes the PCoA score of LDEW group, while the red triangle denotes the PCoA score of LWED group

## Application

In this section, we apply the proposed method into two microbiome studies. The first study includes five groups. We regroup them to construct two data sets with different levels of heterogeneity. In the second study, we link microbiome compositions with two different outcomes.

**Fig. 5:**
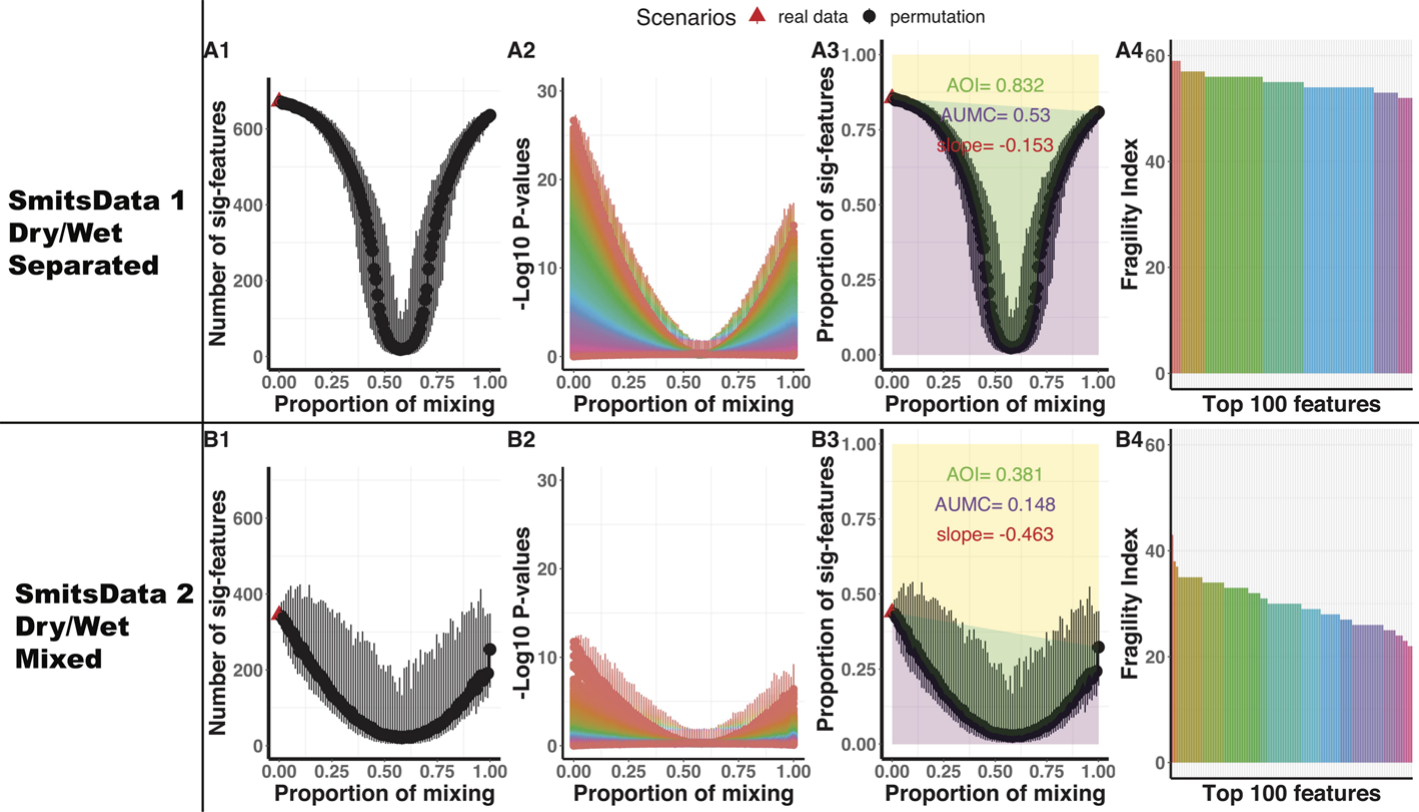
Result comparisons of regrouped data SmitsData 1 (**A1**–**A4**) and SmitsData 2 (**B1**–**B4**) with different levels of heterogeneity. **A1** and **B1** plot the U-Curve of number of significant hits. In **A2** and **B2**, we rank the significance of the 786 features, and then plot their $-\,\log _{10} p$-values with the same order across permutation scenarios. **A3** and **B3** plot the U-Curve of proportion of significant hits. **A4** and **B4** plots the fragility index of the top 100 features with a decreasing order. To save space, the legend listing the names of the 100 features are omitted

**Fig. 6:**
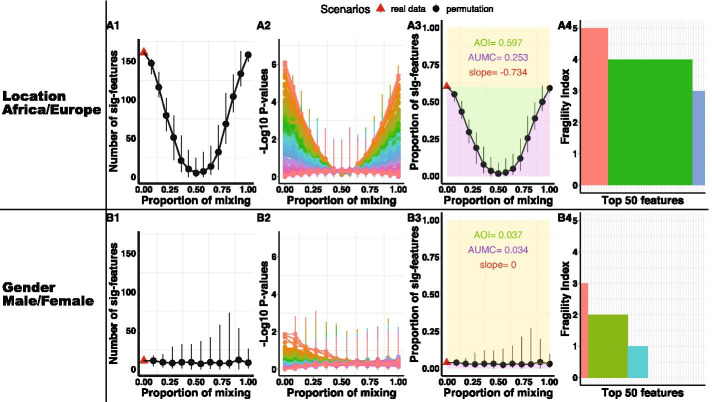
Result comparisons when linking microbiome compositions with location (**A1**–**A4**) and gender (**B1**–**B4**). A1 and B1 plot the U-Curve of number of significant hits. In A2 and B2, we rank the significance of the 267 features, and then plot their $-\,\log _{10} p$-values with the same order across permutation scenarios. A3 and B3 plot the U-Curve of proportion of significant hits. A4 and B4 plots the fragility index of the top 50 features with a decreasing order. To save space, the legend listing the names of the 50 features are omitted

The first study examined the gut microbiota of 350 stool samples collected longitudinally for more than a year from the Hadza hunter gatherers of Tanzania. The samples were collected subsequently with 5 seasonal groups: 2013-LD (Late dry), 2014-EW (Early wet), 2014-LW (Late wet), 2014-ED (Early dry) and 2014 LD (Late Dry). Smits SA, et al. [24] found that Hadza gut microbial community compositions are cyclic and differential by season. They observed that samples from the dry seasons were distinguishable from the wet seasons and were indistinguishable from other dry seasons in sequential years. We combine 2014-ED ($n=33$) and 2014-LD ($n=133$) as the “Dry” group, and combine 2014-EW ($n=62$) and 2014-LW ($n=58$) as the “Wet” group. We call this regrouped data as SmitsData 1. In the same way, we combine 2013-LD ($n=64$) and 2014-EW ($n=62$) as the “LDEW” group, and combine 2014-LW ($n=58$) and 2014-ED ($n=33$) as the “LWED” group. We call this regrouped data as SmitsData 2. We expect that SmitsData 1 is more differential between Dry and Wet group than SmitsData 2 between LDEW and LWED group. As shown in PCoA plots of both data (Fig. 4), the Dry and Wet groups in SmitsData 1 (*p*-${\text {value}}=1e{-}5$ based on PERMANOVA) are more differential than the groups in SmitsData 2 (*p*-${\hbox {value}}=2e{-}5$ based on PERMANOVA).

In total, we have 786 taxonomic features. We perform the progressive permutation tests on SmitsData 1 (Dry $n_1=166$ vs. Wet $n_2=120$) and SmitsData 2 (LDEW $n_1=126$ vs. LWED $n_2=91$). The results of SmitsData 1 (A1–A4) and SmitsData 2 (B1–B4) are shown in Fig. 5. In the observed data (no permutation), differential tests provide more significant hits (*p*-value less than 0.05) from SmitsData 1 (672 in A1) than SmitsData 2 (345 in B1). There are more $-\,\log _{10} p$-values greater than $-\,\log _{10} 0.05$ (A2 vs. B2). The U-Curve of SmitsData 1 (AUMC is 0.53) is steeper than SmitsData 2 (AUMC is 0.148). Based on the plot of fragility index, the overall robustness of the top 100 features from SmitsData 1 (average fragility index is 54.93 in A4) is more than SmitsData 2 (average fragility index is 29.93 in B4). The full permutation scenario for SmitsData 1 is $K_f=70$. So the average scaled fragility index for SmitsData 1 is 0.785. The full permutation scenario for SmitsData 2 is $K_f=53$. So the average scaled fragility index for SmitsData 2 is 0.565. In addition, the initial slopes of the first points for SmitsData 1 and SmitsData 2 are − 0.153 and − 0.463 respectively, which also indicate the significance in SmitsData 1 is more robust. All these results demonstrate that the progressive permutation results can convey and quantify the overall association which is disturbed by heterogeneity. When it comes to feature identification, the proposed method obtains 656 features for SmitsData 1 and 271 features for SmitsData2.

The second study investigated the impact of diet by comparing the gut microbiota of 14 children aged 1–6 years in a village of rural Africa with the gut microbiota of 15 European children of the same age. De Filippo et al. [25] found significant differences in gut microbiota between the two groups, as children at these two locations have different dietary habits. 11 of them are female. 18 of them are male. There is almost no difference in microbiome compositions by gender. In total, we have 267 taxonomic features in the DeFilippo Data. We perform the progressive permutation tests to associate microbiome compositions with location and gender respectively. The results of location (A1–A4) and gender (B1–B4) are shown in Fig. 6. In the observed data, differential tests provide more significant hits for Location (161 in A1) than for Gender (11 for A2). The results illustrate that microbiome compositions are strongly associated with location instead of gender, because AUMC of location (0.253 in A3) is greater than AUMC of gender (0.035 in B3). The U-Curves of gender (B1 and B3) are almost flat, which imply that the overall association between microbiome compositions and gender is weak. Based on the plot of fragility index, the overall robustness of the top 50 features for Location (average fragility index is 4.12 in A4) is more than Gender (average fragility index is 0.98 in B4). The full permutation scenario for Location is $K_f=7$, and the average scaled fragility index for location is 0.589. The full permutation scenario for gender is $K_f=7$, and the average scaled fragility index for gender is 0.14. In addition, the average slopes of the first 7 points for location and gender are − 1.17 and − 0.03 respectively, which also indicate there is no significance for gender across all the scenarios. All these results demonstrate that the progressive permutation method can measure and rank the overall association between microbiome and multiple outcomes of interest. For the outcomes with high association, we will continue to identify the microbiome features that are linked to them.

**Fig. 7:**
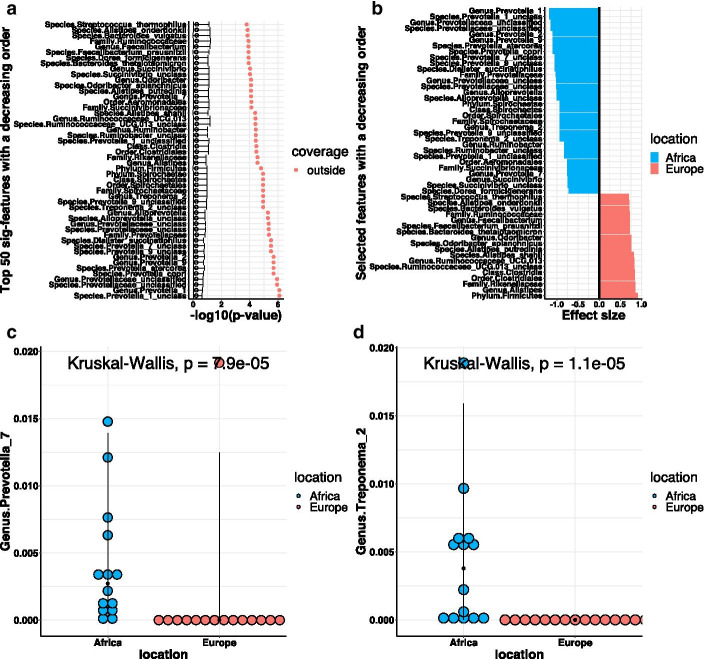
List of discoveries, effect sizes and individual abundances. **a** Denotes the coverage plot of the top 50 features with decreasing order. The color dots denote the $-\,\log _{10} p$-value of top 50 features in the original data (permutation proportion is 0). The horizontal bars describe the 95% quantile confidence intervals of the $-\,\log _{10} p$-value in the full permutation scenario. **b** Denotes the effect sizes of identified features. **c**, **d** Denote the dot plot of abundance of Prevotella and Treponema with median-quantile vertical lines

We include the identification of individual features in our software by observing whether the $-\,\log _{10} p$-values of targeted features lie within the 95% confidence interval of median $-\,\log _{10} p$-values of the full permutation scenario. The proposed method has identified 155 features for location and 0 features for gender. As shown in the upper left panel in Fig. 7, all the top 50 features are significant. The effect sizes of these 50 significant features are plotted in the upper right panel. Our findings are consistent with published results [25]. Firmicutes are more abundant in European children than in African children. Prevotella and Treponema (Spirochaetaceae) are more abundant in African children than in European children (as shown in the lower panels of Fig. 7).

In summary, our method first explores the overall association (that might be complicated by heterogeneity) between microbiome compositions and outcome variable. If the association is reasonable, it will identify the significance of individual hits, list their effect sizes and plot individual abundances.

## Analytical property

Various summary statistics, like mean, variances, median and rank sums, have been used to analyze differences between two groups. Each statistic goes along with an assumption of a sample distribution, including normal, negative binomial and so on. Among these, the mean test under a normal assumption is one of the most widely-used statistical techniques for group comparisons. Other types of tests extend the standard to broader situations that require specific assumptions or less restrictions. Therefore, it is worthwhile to pursue the theoretical aspects of the progressive permutation method in a basic setup that performs Z-tests. The theoretical results from parametric tests can provide insights to the progressive permutation coupling non-parametric tests, as we expect to observe similar patterns between them. To simplify the problem, we assume observing two groups of variables from Gaussian family. Both groups have the same number of variables *p*. The population distribution of Group 1 is ${\mathcal {N}}(\mu _j^1,\sigma ^2)$, and the population distribution of Group 2 is ${\mathcal {N}}(\mu _j^2,\sigma ^2)$. We aim to test the hypothesis $H_{0j}: \mu _j^1=\mu _j^2,\, {\mathrm{versus}}\, H_{1j}: \mu _j^1\ne \mu _j^2$.

For the sample data, we use $x_{ij}^1$ to denote the *i*th observation of the *j*th variable in Group 1 and $x_{ij}^2$ to denote the *i*th observation of the *j*th variable in Group 2. The data samples are generated from Gaussian distributions with $x_{ij}^1\sim {\mathcal {N}}(m_j^1,\sigma ^2)$ and $x_{ij}^2\sim {\mathcal {N}}(m_j^2,\sigma ^2)$. The observations of every variable in each group are independent and identically distributed. We denote the grouping labels in Group 1 as $I^1=\{1,\ldots , n_1\}$. We denote the grouping labels in Group 2 as $I^2=\{1,\ldots , n_2\}$. To test the population mean difference ($\mu _j^1-\mu _j^2$) of the *j*th variable between the two groups, we calculate the sample mean difference as below:

2 $$\begin{aligned} {{\varvec{\bar{x}}}}_j^1-{{\varvec{\bar{x}}}}_j^2=\frac{1}{n_1}\sum _{i=1}^{n_1} x_{ij}^1-\frac{1}{n_2}\sum _{i=1}^{n_2} x_{ij}^2\sim {\mathcal {N}}\left(m_j^1-m_j^2,\frac{n_1+n_2}{n_1n_2}\sigma ^2\right). \end{aligned}$$

Now we perform the progressive permutation method and randomly draw *k* samples from group 1 and *k* samples from group 2, and then exchange their grouping labels. We denote the selected labels in Group 1 as $I_k^1=\{i^1_1,\ldots , i^1_k\}$. We denote the selected labels in Group 2 as $I_k^2=\{i^2_1,\ldots , i^2_k\}$. Then the sample mean difference of the *j*th variable in permutation scenario *k* becomes

3 $$\begin{aligned} {{\varvec{\bar{x'}}}}_j^1-{{\varvec{\bar{x'}}}}_j^2&=\frac{1}{n_1}\sum _{i\in I^1\setminus I^1_k} x_{ij}^1 + \frac{1}{n_1}\sum _{i\in I^2_k}x_{ij}^2 - \frac{1}{n_2}\sum _{i \in I^2\setminus I^2_k} x_{ij}^2 - \frac{1}{n_2}\sum _{i\in I^1_k}x_{ij}^1\\ &\sim {\mathcal {N}}\left( \left(1-\frac{n_1+n_2}{n_1n_2}k\right)\left(m_j^1-m_j^2\right),\frac{n_1+n_2}{n_1n_2}\sigma ^2\right) . \end{aligned}$$

We assume $m_j^1>m_j^2$. The sample mean differences after permutation (3) are smaller than those before permutation (2). Denote $\delta _j=\frac{m_j^1-m_j^2}{\sigma }$. The *p*-value of the *j*th variable (under null hypothesis) is

4 $$\begin{aligned} p_j(k)&= P\left( |z|>\frac{({{\varvec{\bar{x'}}}}_j^1-{{\varvec{\bar{x'}}}}_j^2)-(\mu _j^1-\mu _j^2)}{\sqrt{\frac{n_1+n_2}{n_1n_2}\sigma ^2}}\bigg \vert H_{0j}:\mu _j^1-\mu _j^2=0\right) \\&=2P\left( z+\frac{{{\varvec{\bar{x}}}}_j^1-{{\varvec{\bar{x}}}}_j^2}{\sqrt{\frac{n_1+n_2}{n_1n_2}\sigma ^2}}<0\right) \\&= 2\Phi \left( -\sqrt{\frac{n_1n_2}{2(n_1+n_2)}}(1-\frac{n_1+n_2}{n_1n_2}k)\delta _j\right) , \end{aligned}$$

where $k\le \lceil \frac{n_1n_2-1}{n_1+n_2+2} \rceil$. $\Phi (\cdot )$ denotes the cumulative function of standard normal distribution. Therefore, with the increase of exchanged labels *k*, $-\,\log _{10} p$-value is smaller. As we perform two sided Z-tests in each scenario, the permutation results (*p*-values) are symmetric with respect to the fully mixing scenario $K_f=\lceil \frac{n_1n_2-1}{n_1+n_2+2} \rceil$. Then we can obtain the *p*-value of the *j*th variable when $k=K_f,\ldots , K$ as $p_j(k)=2\Phi \left( \sqrt{\frac{n_1n_2}{2(n_1+n_2)}}\left(1-\frac{n_1+n_2}{n_1n_2}k\right)\delta _j\right)$. $-\,\log _{10} p_j(k)$ decreases with *k* when $0\le k\le K_f$ and increases with *k* when $K_f\le k\le K$.

**Fig. 8:**
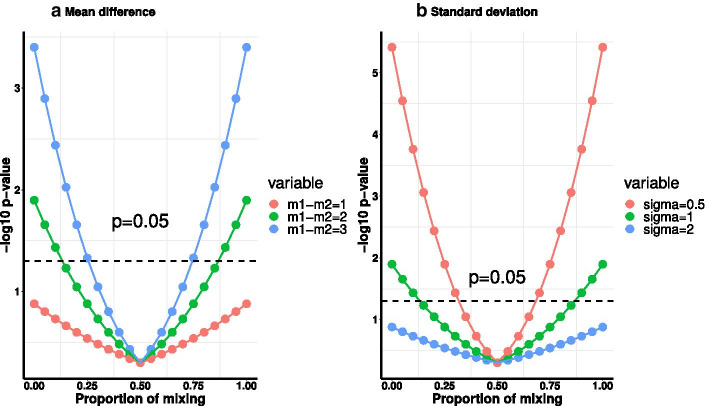
U-Curve plots of *p*-values calculated from formula (4). Both of the sample sizes $n_1$ and $n_2$ are 20. x-axis is $\frac{k}{K}$. In **a**, standard deviation $\sigma$ is fixed at 2. In **b**, mean difference $m_j^1-m_j^2$ is fixed at 1

For real-world data, the scaled sample mean difference $\delta _j$ takes a series of different numbers. For example, suppose that $n_1=n_2=n$ and $\delta _j$ ranges from 0 to 2, then for $k=0$, the *p*-values $p_j(0)=2\Phi (-\frac{\sqrt{n}}{2}\delta _j)$ will be distributed uniformly between 0 and 1. If we assume an extreme case that all the sample mean differences are the same and equal to 0 ($\delta _j=0$), indicating there is no group difference, all the *p*-values will be 1 across all permutation scenarios so that the curve of $-\,\log _{10} p$-values and number of significant features will become a flat horizontal line at 0. We define $\frac{k}{K}$ as the proportion of mixing. We let $n_1=n_2=20$. If we generate the sample data with group difference meaning that $\delta _j>0$, then we can observe in Fig. 8, $-\,\log _{10} p_j(k)$ is a U-Curve of $\frac{k}{K}$. To simplify the the visualization, we assume all the $\delta _j$ are the same, so then the *p*-values are the same as well. If the differences of sample means are bigger, the U-Curve is steeper. If the standard deviation of the samples is bigger, the U-Curve is flatter. Therefore, the shape of the U-Curve measures how differential the quantifies of interest are between the two groups.

## Discussion

In this work, we propose a method for the analysis of microbiome data which progressively permutes a grouping factor and performs differential abundance tests in each scenario. To convey the overall association with the grouping factor, we summarize the resulting *p*-values by the number of significant hits. This number will exhibit a U-Curve across mixing depths if the overall association between the microbiome and the grouping factor is not zero. The AUMC provides a summary of the progressive permutation results, allowing for quantification of the overall signal strength, which is interestingly impacted by heterogeneity. Simulation results show that the shape of the U-Curve can quantify different levels of heterogeneity within data sets. If we have multiple grouping factors, we can rank their AUMCs by associating each grouping factor with the microbiome composition as a whole. In general, we recommend focusing on grouping factors with higher AUMC values for subsequent in-depth analysis.

Once we have decided on a grouping factor of interest, we may then seek to identify microbiome features which are robustly associated with the grouping factor. Based on the permutation results, we can rank all the microbiome features by their fragility index, where larger values of the fragility index correspond to more robust discoveries. We can identify potentially relevant microbiome features by comparing the *p*-values of the observed data with the confidence region of *p*-values for the fully mixed data. The simulation and real data application show that our proposed method can convey the overall association between microbiome compositions and outcomes of interest, rank the robustness of the discovered features, and identify robust individual hits.

Through simulations, we show that the signal strength of the observed features is controlled by several factors, including the proportion of zeros, mean difference, and variance. The correctness of the ordering of the signals is partly affected by the choice of test used to obtain *p*-values within each permutation setting. The Wilcoxon test is a nonparametric test, which takes into account the ranks of the abundances for each taxa. Although the ordering results are not perfect, we show through simulations that the proposed method can identify the differential features with a high accuracy rate. Our paper is mainly designed for the general exploration and visualization of microbiome data, and does not come with a formal inference method. The measures we propose, such as AOI or AUMC, are meant to be descriptive, but researchers could take the results generated from our method as a guide to help with identifying robust features. At this time, our method does not control the false discovery rate or calculate adjusted *p*-values. In future work, we will consider using the progressive permutation results to adjust the *p*-values by controlling the empirical Bayes false discovery rates.

To better understand the relationship between progressive permutation and hypothesis testing, we use the language of signal and noise to describe hypothesis testing. The null hypothesis can be identified as the case where the data contain only noise and no signal. The alternative hypothesis is the case where the data contain both important signals and noise [26]. Progressive permutation progressively mixes the samples between two groups. With each increase of mixing, the proportion of signal decreases, while the proportion of noise increases. Therefore, the fully permuted data can be considered as realizations of the null hypothesis, while the observed data (without permutations) can be considered as a realization of the alternative hypothesis. Conceptually, progressive permutation connects the binary ends of hypothesis testing from the alternative hypothesis to the null hypothesis in a continuous manner. Therefore, the proposed method considers the signal identification problem as progressively singling out signals from permuted randomized versions of an original data set.

In this paper, we focus on linking microbiome composition with a binary outcome, creating a new framework to understand the significance and robustness of microbiome features. Following the same logic, we can extend the binary outcome to a continuous outcome. When constructing the progressive permutation scenarios, we permute a proportion (select *k* samples and calculate $\frac{k}{n}$) of the continuous outcome. In each scenario, we perform Kendall’s tau and Spearman’s rank correlation tests to associate microbiome compositions with the permuted continuous outcome. We then adopt similar procedures as in the binary outcome to summarize the permutation results. We have applied the progressive permutation with a continuous outcome to a sample data set (see Additional file 1: Sect. S5).

We have developed these methods into user-friendly and efficient R Shiny tools with visualizations. In our implementation, we first perform differential testing of each feature, and then obtain the $-\, \log _{10}p$-values from permutations of the data. By default, we use the Wilcoxon rank sum test to compute the *p*-values, since it is a robust nonparametric test. Our proposed method can also utilize *p*-values obtained from other testing methods, such as DESeq. This demonstrates the great potential of the progressive permutation method to be extended to new settings.

## Supplementary Information

**Additional file 1. Section S1**: Mathematical notations. **Section S2**: Computational time. **Section S3**: Distribution of zeros. **Section S4**: Results of two permutation methods. **Section S5**: Results of continuous outcome.

## Data Availability

Smits data were provided in association with the following publication: Smits et al. “Seasonal cycling in the gut microbiome of the Hadza hunter-gatherers of Tanzania”, *Science*. 2017;357(6353):802–806. The 16S rRNA amplicon sequence data and shotgun metagenomic data have been deposited in the Sequence Read Archive (SRA) under the project IDs PRJNA392012, PRJNA392180 (www.ncbi.nlm.nih.gov/sra). DeFilippo data were provided in association with the following publication: De Filippo et al. “Impact of diet in shaping gut microbiota revealed by a comparative study in children from Europe and rural Africa”, *Proceedings of the National Academy of Sciences*. 2010;107(33):14691–14696. The 16S rRNA amplicon sequence data were submitted to the Sequence Read Archive (SRA) using ISA tools (ISAcreator and ISAconverter, http://isatab.sourceforge.net/index.html). The dataset is available at http://www.ebi.ac.uk/ena/data/view/ERP000133. In addition, RShiny App is accessible at https://biostatistics.mdanderson.org/shinyapps/ProgPerm. R codes and example data are available at https://github.com/LyonsZhang/ProgPerm.

## Abbreviations

AOI: Area of interest
AUMC: Area under the mixing curve
