# Supplementary material for: ProgPerm: Progressive permutation for a dynamic representation of the robustness of microbiome discoveries
Source: BMC Bioinformatics. 2021 Mar 17;22:126. doi: 10.1186/s12859-021-04061-3 (PMC7972227; doi:10.1186/s12859-021-04061-3)
Supplement: Supplementary file 1 — Additional file 1. Section S1: Mathematical notations. Section S2: Computational time. Section S3: Distribution of zeros. Section S4: Results of two permutation methods. Section S5: Results of continuous outcome. [file 12859_2021_4061_MOESM1_ESM.pdf]

Supplementary Material for "Progressive permutation for a dynamic representation of the robustness of microbiome discoveries"

Liangliang Zhang[1], Yushu Shi[2], Kim-Anh Do[1], Christine Peterson[1] and Robert Jenq[3]
[1]*Department of Biostatistics, University of Texas MD Anderson Cancer Center, Houston, Texas, U.S.A.*
[2]*Department of Statistic, University of Missouri, Columbia, Missouri, U.S.A.*
[3]*Department of Genomic Medicine, University of Texas MD Anderson Cancer Center, Houston, Texas, U.S.A.*

S1. MATHEMATICAL NOTATIONS

Theorem 1. *If n_1 and n_2 are non-negative integers, then we have $\sum_{k=0}^{K}\binom{n_1}{k}\binom{n_2}{k} = \binom{n_1+n_2}{K}$, where $K = \min(n_1, n_2)$.*

Proof. Without losing any generality, we assume $n_1 < n_2$, then $K = n_1$. As $(1+x)^{n_1}(1+x)^{n_2} = (1+x)^{n_1+n_2}$, then we can obtain $\sum_{k=0}^{n_1}\binom{n_1}{n_1-k}\binom{n_2}{k}x^{n_1-k}x^k = \binom{n_1+n_2}{n_1}x^{n_1}$. We let $x = 1$ and $n_1 = K$, the fact of $\binom{n_1}{n_1-k} = \binom{n_1}{k}$ will complete the proof. □

Theorem 2. *Suppose n_1, n_2 and k are non-negative integers. The combination coefficient $\binom{n_1}{k}\binom{n_2}{k}$ approaches its maximum, when k equals the closest integer greater than $\frac{n_1 n_2 - 1}{n_1+n_2+2}$.*

Proof. To obtain the maximum of $C(k) = \binom{n_1}{k}\binom{n_2}{k}$, we need to find a k such that $C(k+1) \leq C(k)$. As we know that $C(k+1) = \binom{n_1}{k+1}\binom{n_2}{k+1} = \frac{(n_1-k)(n_2-k)}{(k+1)(k+1)}C(k)$, then we have $\frac{(n_1-k)(n_2-k)}{(k+1)(k+1)} < 1$. We obtain $k > \frac{n_1 n_2 - 1}{n_1+n_2+2}$, which completes the proof. □

S2. COMPUTATIONAL TIME

We describe the computational burden as follows. In each permutation scenario, we have ν draws. In each draw, we perform p independent tests. If we add the number of tests across the all the permutation scenarios, we have

$$\begin{aligned}\sum_{k=1}^{K}\nu p &= Np\sum_{k=1}^{K}\left(\log\binom{n_1}{k} + \log\binom{n_2}{k}\right)\\ &\leq Np\log\left(\sum_{k=0}^{K}\binom{n_1}{k}\binom{n_2}{k}\right)\\ &= Np\log\binom{N}{K}\\ &\leq NpK(1+\log N - \log K)\end{aligned}$$

If the two groups have the same sample size, meaning $n_1 = n_2$, then $K = N/2$. The time complexity will be less than $0.85 \times pN^2$. For example, if the sample size N is 100, the number of microbiome features p is 1000, the time of running a Wilcoxon Rank Sum Test is 0.004 seconds, then the total time of executing the progressive permutation will be 9 hours. However, utilizing the parallel computing (implemented via the "doParallel" R package) on an 8-core computer produces a running time of 30 minutes.

S3. DISTRIBUTION OF ZEROS

Zero-Inflation is one of the main characteristics of microbiome data. Figure S1 shows the distribution of zeros across samples and variables of SimData 1, SimData 2 and SimData 3. The distribution is comparable to the distribution of zeros in DeFilippo Data S2.

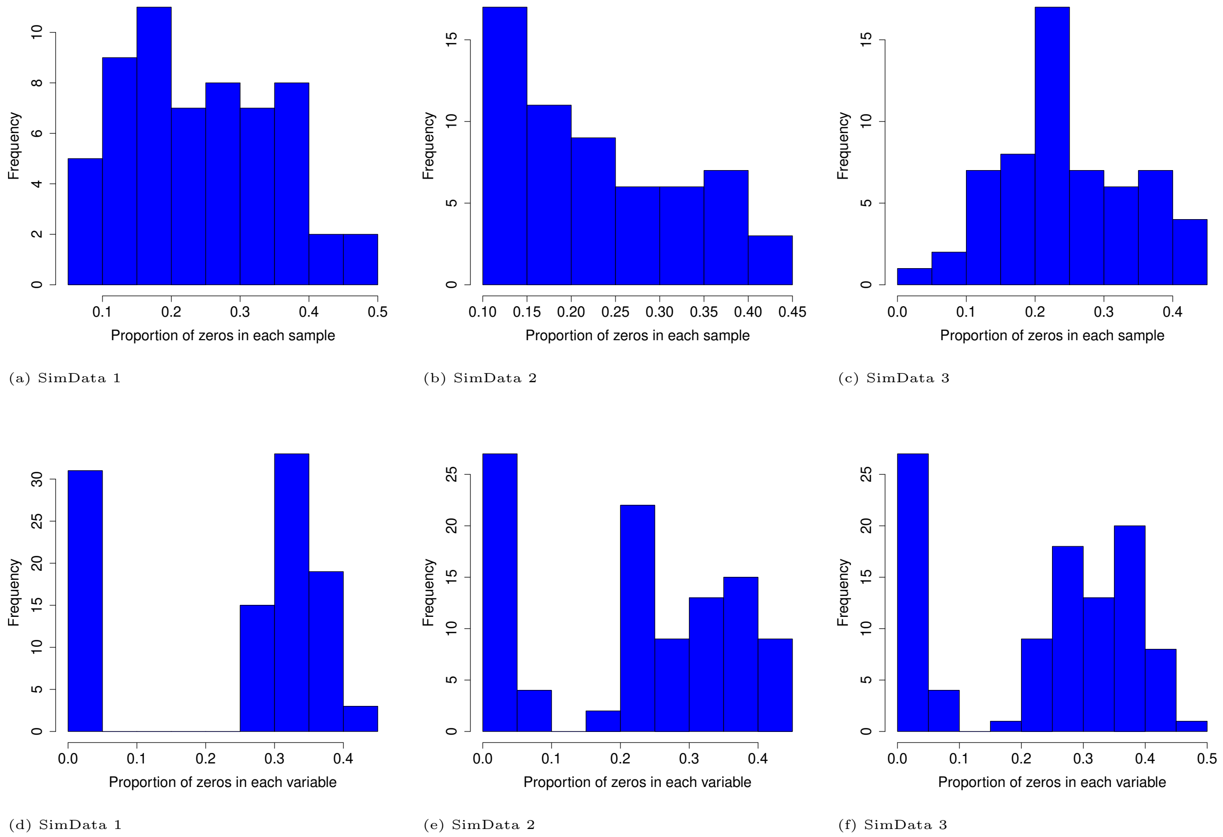

(a) SimData 1 (b) SimData 2 (c) SimData 3

(d) SimData 1 (e) SimData 2 (f) SimData 3

FIG. S1: The distribution of zeros across samples and variables of simulated data sets.

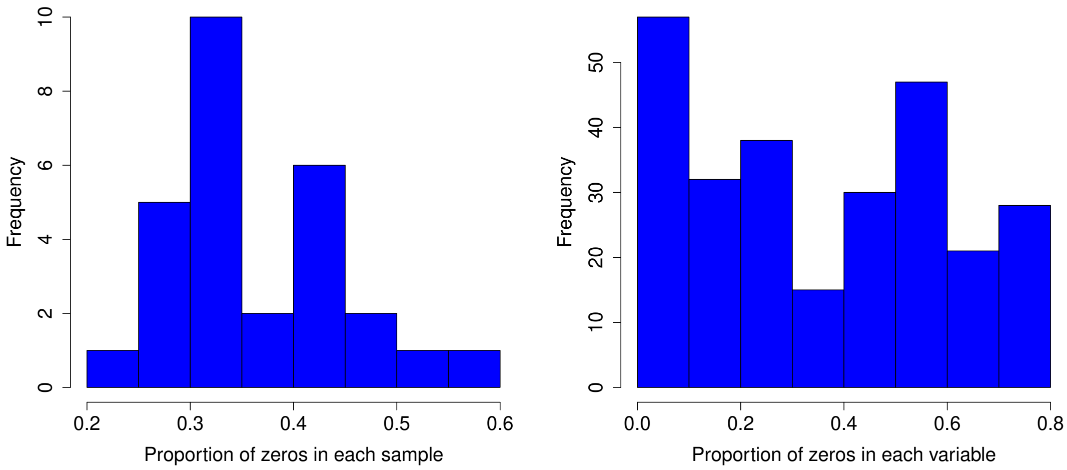

FIG. S2: The distribution of zeros across samples and variables of DeFilippo Data.

S4. RESULTS OF TWO PERMUTATION METHODS

In this section, we provide the results of the permutation method with Wilcoxon test and DESeq. We plot the traces of $-\log_{10} p$-values in Figure S3, S5, S7, and S9. We plot the number of significant features in Figure S4, S6, S8, and S10. In general, the traces of p-values from DESeq method spread out more (with a wider range) than the traces of p-values from the Wilcoxon test. For setting 1, the number of significant features does not approach zero in the full permutation scenario when using the permutation method with DESeq.

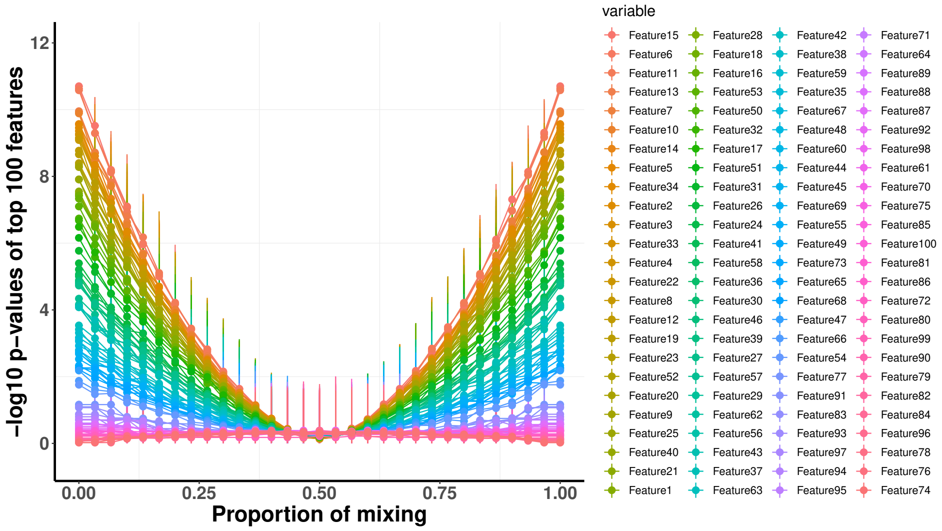

(a) Permutation with Wilcoxon test

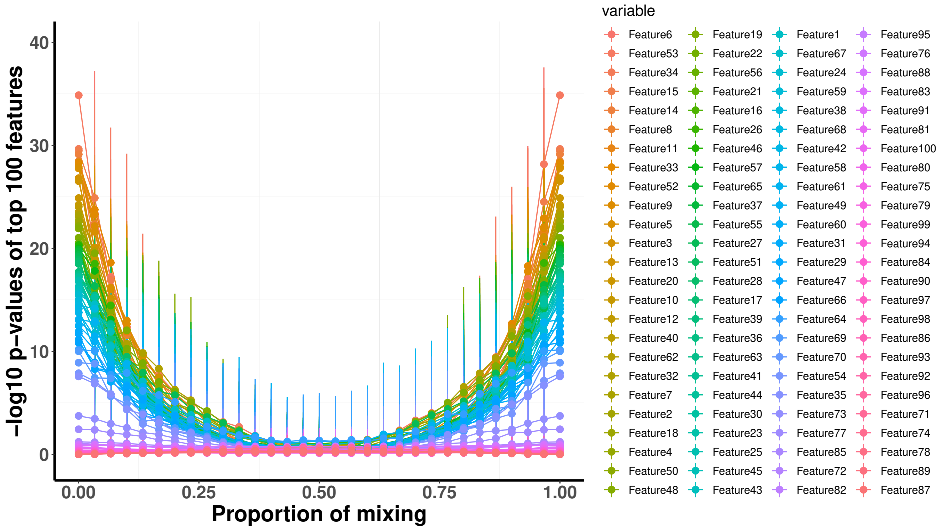

(b) Permutation with DESeq

FIG. S3: **Set 1 with dense signal.** Plot of traces of $-\log_{10} p$-values vs. proportion of mixing. Both the methods are implemented on simulation data Set 1, which varies the zero inflation parameters for each variable. The data contains a dense signal, where the number of true differential features is 70.

S5. RESULTS OF CONTINUOUS OUTCOME

In medical research, continuous outcomes, such as BMI, clinical scores, are available and linked with human microbiome. However, "the curse of dimensionality" can also be confronted when we use a canonical multiple linear

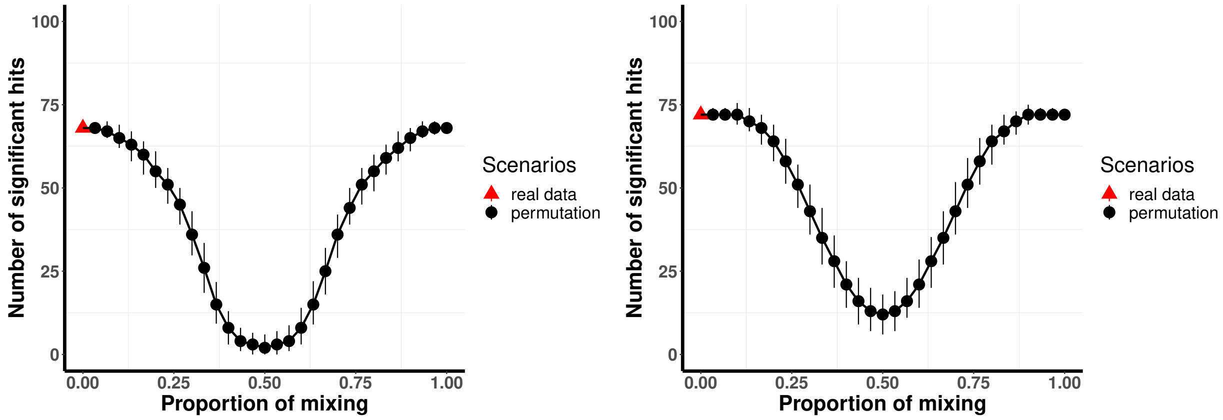

(a) Permutation with Wilcoxon test

(b) Permutation with DESeq

FIG. S4: **Set 1 with dense signal.** Plot of number of significant hits vs proportion of mixing. Both methods are implemented on simulation data Set 1, which varies the zero inflation parameters for each variable. The data contains a dense signal, where the number of true differential features is 70.

regression to estimate the associations. More taxa would be identified as significantly associated with outcome variable, if more OTUs were generated and included in linear models. Because the coefficient of determination R^2 (R is multiple correlation between outcome and regressors) is increasing with the number of regressors in the model [1]. Besides, the sign and amount of coefficient estimates are usually messed up by heterogeneity of the data. With these similar motivations in the binary response case, we extended the progressive permutation for a continuous outcome.

We describe the progressive permutation procedure for a continuous outcome as follows. Suppose we collect N samples of microbiome specimens and obtain p microbiome features. We use $k = \{0, 1, \cdots, K\}$ to describe the progressive permutation scenarios. $k = 0$ describes the original data without any permutation. We use K to describe the maximal permutation scenario which can be specified by user. For instance, we set up K to be 10 in the following application. At each permutation scenario k, we randomly split the outcome variable into two parts with sample sizes of $n_k^1 = N\frac{k}{K}$ and $n_k^2 = N(1 - \frac{k}{K})$ in each part. We permute the outcome values in the first part and keep the second part unchanged. Then we perform p correlation tests to associate each microbiome feature with the permutated continuous outcome (such as Kendall 's tau or Spearman Rank Correlation tests) and obtain all the p-values. We can calculate the number of significant taxa as $\text{nsig}(k) = \sum_{j=1}^{p} I_{p_j(k) \leq \alpha}$, where α is the prespecified significance level. We expect to see the lowest $\text{nsig}(k)$ in the fully mixing scenario K. The number of significant hits $\text{nsig}(k)$ decreases with the proportion of mixing k/K.

At each permutation scenario k $(1 \leq k < K)$, we start from a random seed and try a subset of $\nu = N \log \binom{N}{n_k^1}$ draws. Therefore, for each variable j, we obtain ν samples of p-values $p_j(k)$ and numbers of significant taxa $\text{nsig}(k)$. We summarize the distribution of these samples by their medians and 2.5%-97.5% quantile intervals. To visualize these p-values in an organized manner, we rank the significance of all the variables in the observed data, and then plot their $-\log_{10} p$-values with the same order across permutation scenarios. In general, the paralleled traces of $-\log_{10} p$-values of more significant variables will be on the top of less significant ones. With the increase of mixing, the significant p-values gradually become nonsignificant, indicating that the signal is weaker and the noise is stronger. As there would be almost no signal if data were fully mixed, all the p-values are close to 1 at the full permutation scenario $k = K$. Therefore, we could observe a decreasing trend in the number of significant features from no permutation scenario $(k = 0)$ to the full permutation scenario $k = K$.

We applied the progressive permutation method to a real data set, which investigates the associations between microbiome and fatigue. The fatigue score ranges from 0 to 10. We treated it as a continuous outcome. The sample data set includes 88 samples and 841 microbial taxa. The generated results are shown as follows. The overall association between microbiome compositions and fatigue is not very strong, since the trace plot of number of significant features is flat (shown in FIG. S11a). There are not many features with -log10(p-value) more than 1.3 (-log10(0.05)) (FIG. S11b). The number of significant features obtained from differential tests on observed data is 35. Most of the effect sizes range between -3 and 3 (FIG. S11c). The correlations range between -0.25 and 0.25 (FIG. S11d), which are weak. The conclusion of weak overall association can be made from FIG. S12, as AOI is 0.031, AUMC is 0.029 and slope is -0.031. FIG. S13 lists all the fragility index of the top 35 significant features (p-value less than 0.05 in observed data) with a decreasing order. Enterococcus faecalis and Enterococcaceae are the two taxa

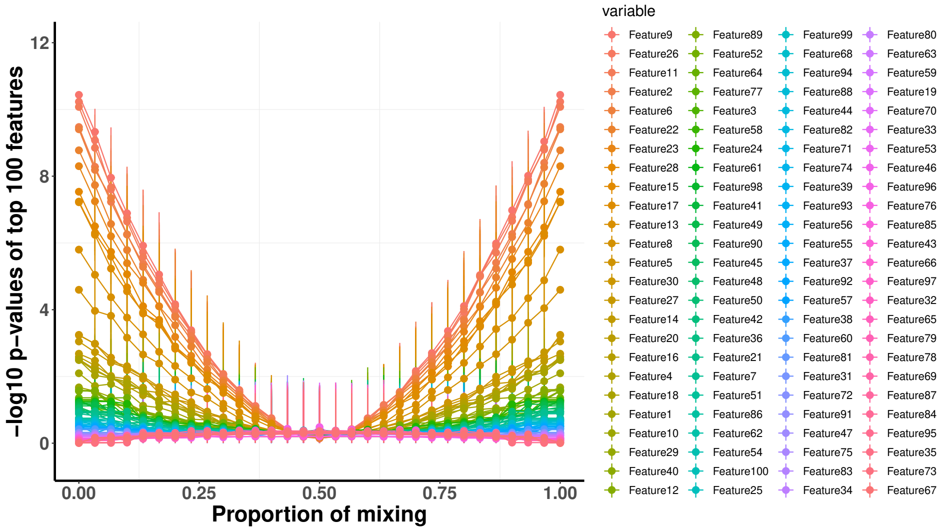

(a) Permutation with Wilcoxon test

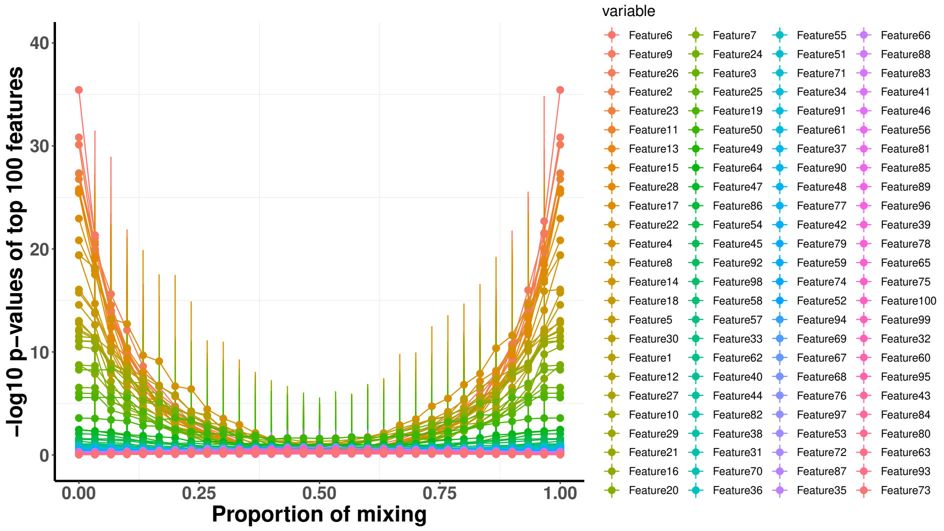

(b) Permutation with DESeq

FIG. S5: **Set 1 with sparse signal.** Plot of traces of $-\log_{10} p$-values vs. proportion of mixing. Both methods are implemented on simulation data Set 1, which varies the zero inflation parameters for each variable. The data contains a sparse signal, where the number of true differential features is 30.

with the highest number. Currently, to identify significant features, we compare the p-values of the original data with the p-values of full permutation scenario (separating signals from random noises). FIG.‘S14 does a coverage plot of the top 35 features with decreasing order and identifies 18 features to be selected ones. FIG. S14 lists the effect sizes of all the 18 selected features. For instance, Enterococcus faecalis is positively associated with fatigue.

In summary, progressive permutation with continuous variable can not only show the overall association with microbiome compositions, but also identify the significance of individual features. Analogously, we could extend the progressive permutation model to other types of outcomes, such as count data, ordinal variable, etc. By choosing proper test statistics for each data type, we could show the similar converging trend of the p-values.

The distribution of p-value is crucial for correction of false positive rates. As we can see in FIG. S16, with increase of mixing proportion, the p-value leaves the area of significance ($p < 0.05$) and redistribute themselves to nonsignificant areas. In other words, p-values changes from a skewed distribution (peak is close to 0) to a uniform distribution. Because the p-value is uniformly distributed when the null hypothesis is true meaning that there is no signal in the full permutation scenario. These information might be useful to adjust p-values according to null distributions.

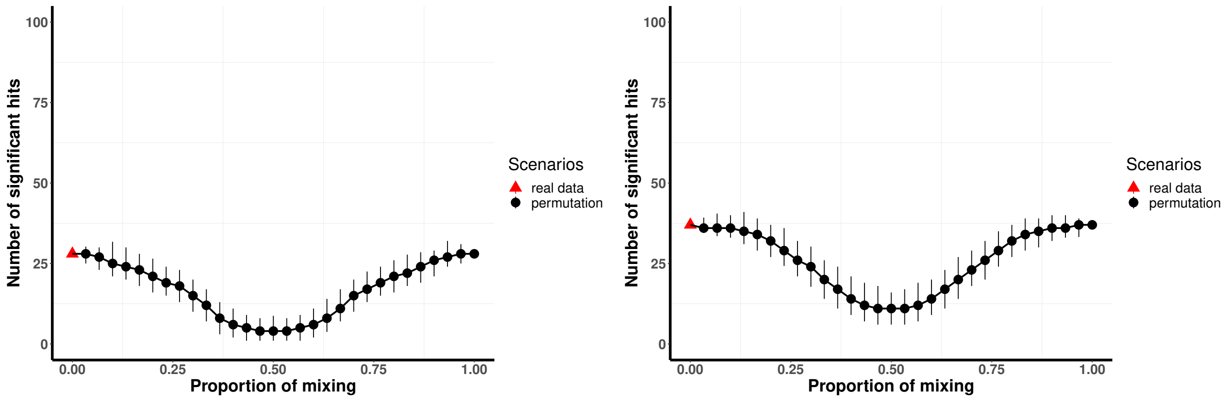

(a) Permutation with Wilcoxon test

(b) Permutation with DESeq

FIG. S6: **Set 1 with sparse signal.** Plot of number of significant hits vs proportion of mixing. Both methods are implemented on simulation data Set 1, which varies the zero inflation parameters for each variable. The data contains a sparse signal, where the number of true differential features is 30.

[1] T. O. Kvålseth, "Cautionary note about R^2," *The American Statistician*, vol. 39, no. 4, pp. 279–285, 1985.

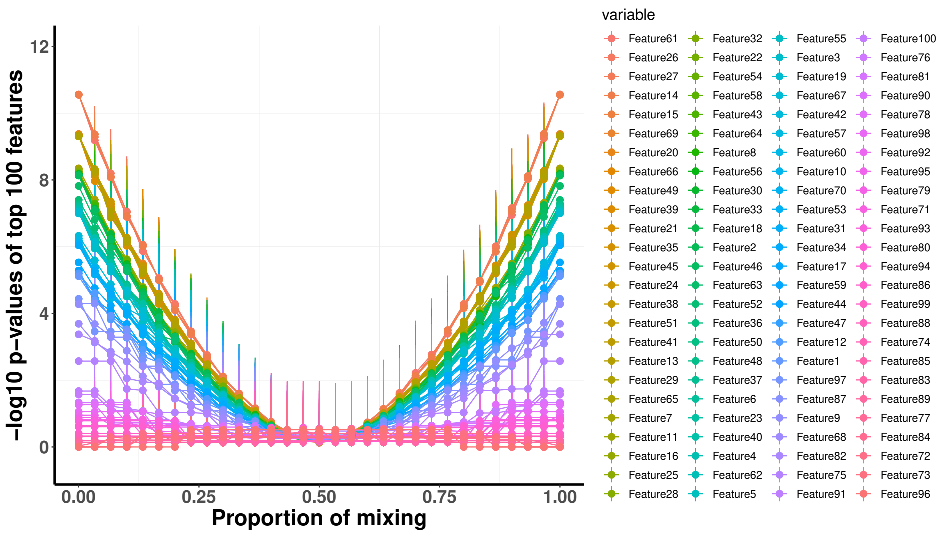

(a) Permutation with Wilcoxon test

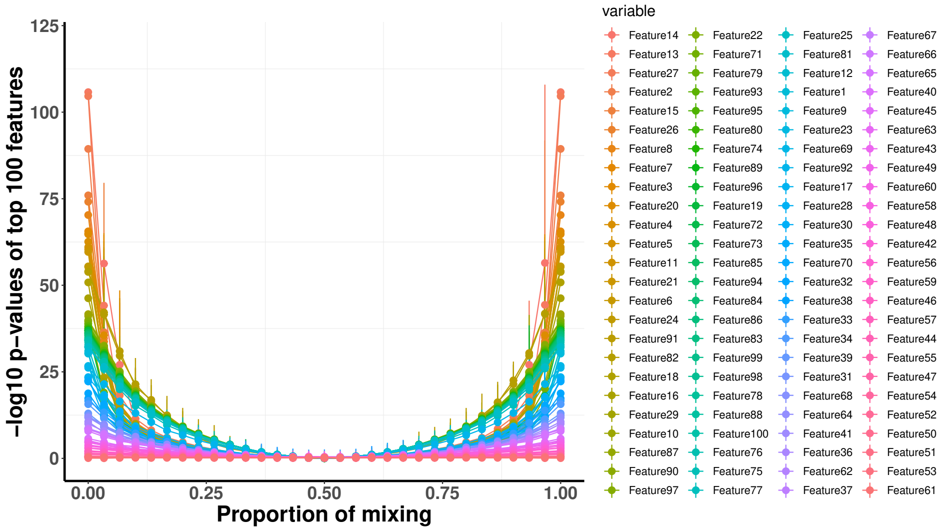

(b) Permutation with DESeq

FIG. S7: **Set 2 with dense signal.** Plot of traces of $-\log_{10} p$-values vs. proportion of mixing. Both methods are implemented on simulation data Set 2, which varies the mean difference parameters for each variable. The data contains a dense signal, where the number of true differential features is 70.

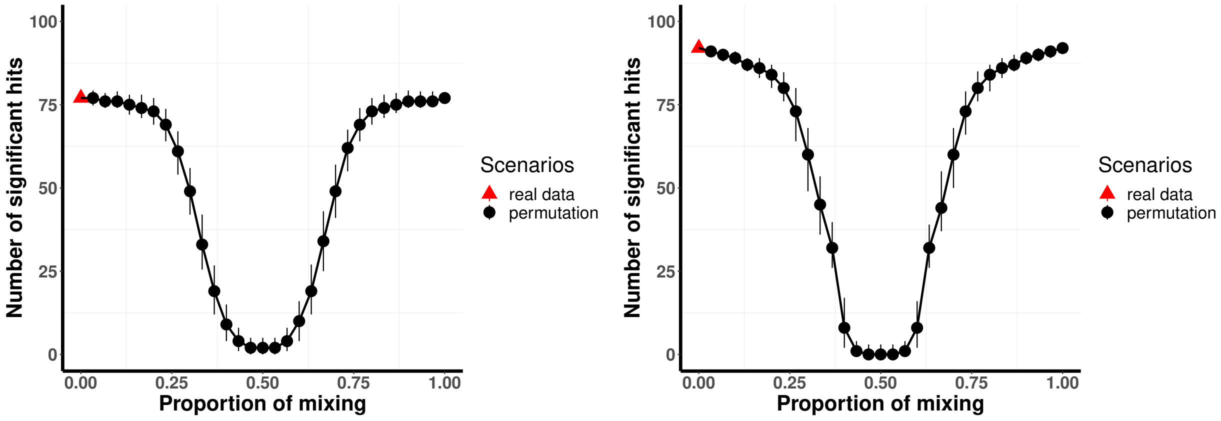

(a) Permutation with Wilcoxon test

(b) Permutation with DESeq

FIG. S8: **Set 2 with dense signal.** Plot of number of significant hits vs proportion of mixing. Both methods are implemented on simulation data Set 2, which varies the mean difference parameters for each variable. The data contains a dense signal, where the number of true differential features is 70.

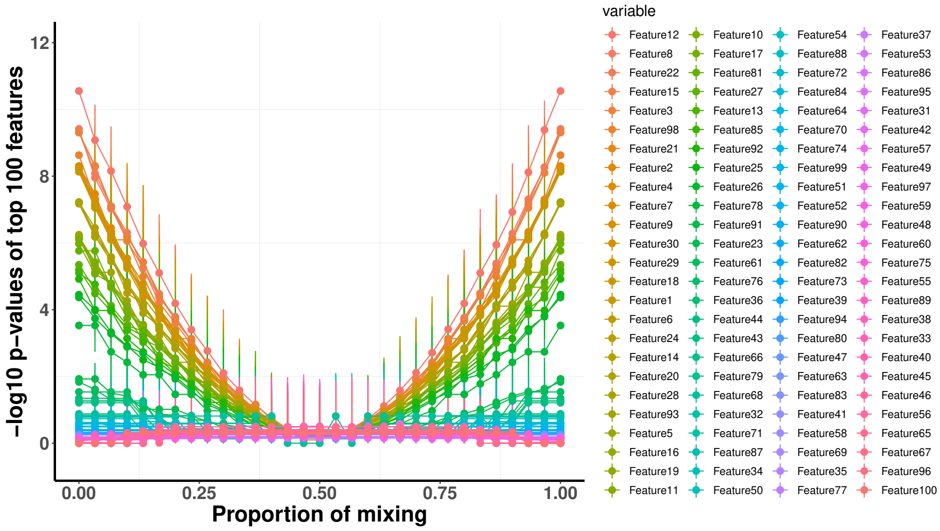

(a) Permutation with Wilcoxon test

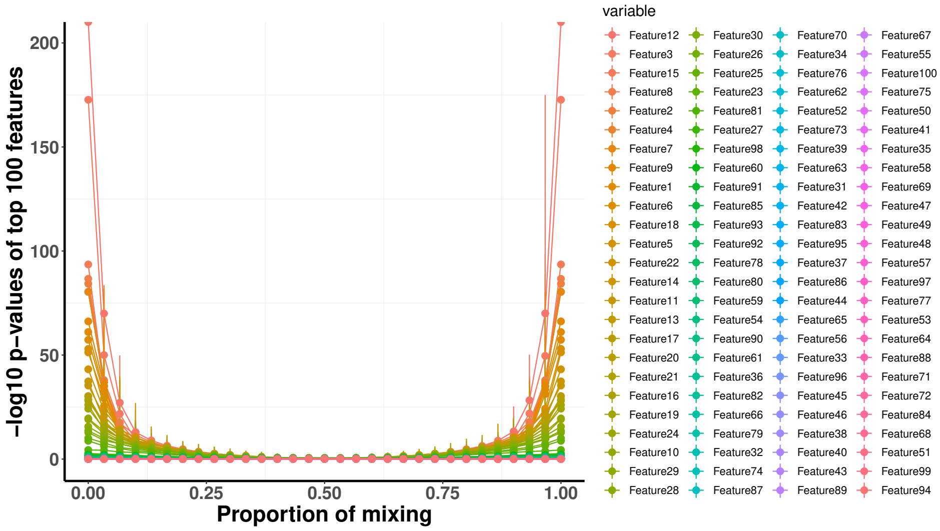

(b) Permutation with DESeq

FIG. S9: **Set 2 with sparse signal.** Plot of traces of $-\log_{10} p$-values vs. proportion of mixing. Both methods are implemented on simulation data Set 2, which varies the mean difference parameters for each variable. The data contains a sparse signal, where the number of true differential features is 30.

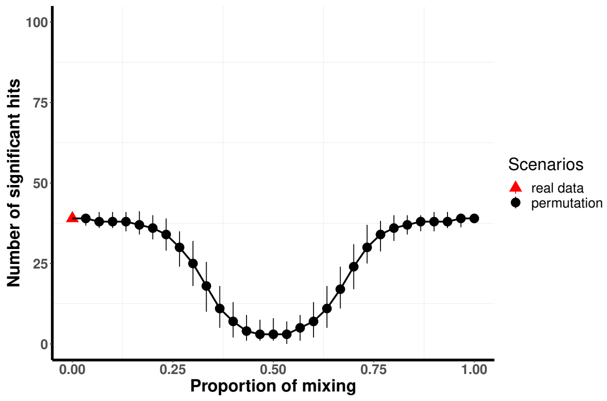

(a) Permutation with Wilcoxon test

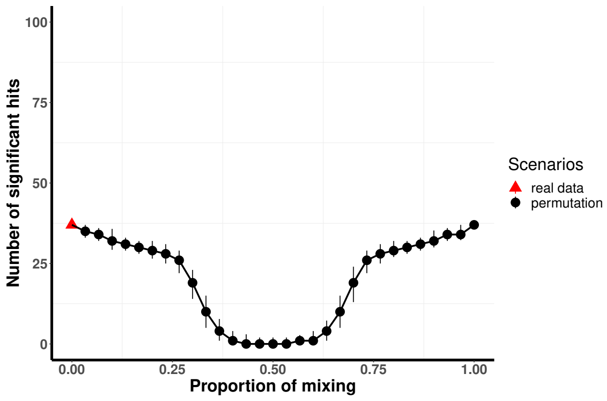

(b) Permutation with DESeq

FIG. S10: **Set 2 with sparse signal.** Plot of number of significant hits vs proportion of mixing. Both methods are implemented on simulation data Set 1, which varies the mean difference parameters for each variable. The data contains a sparse signal, where the number of true differential features is 30.

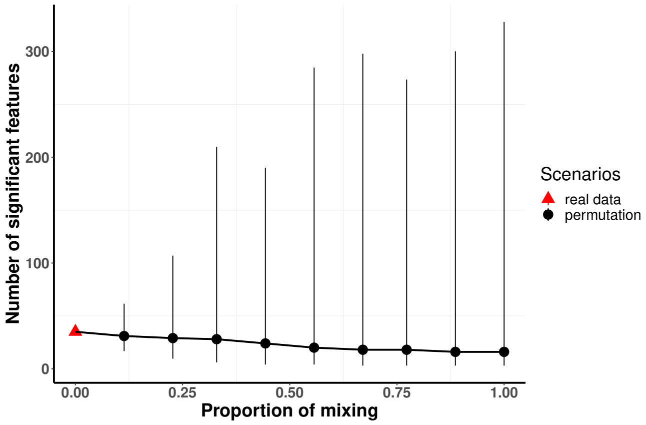

(a) The curve plot showing the decreasing trend of number of significant features across all the permutation scenarios. The red triangle denotes the number of significant features in real data. The black point denotes the median of number of significant features in the permuted data. The vertical line denotes the 2.5%-97.5% quantile intervals of number of significant features in the permuted data.

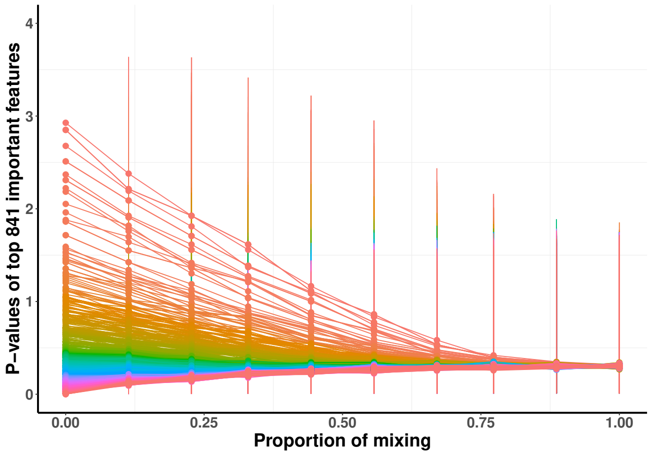

(b) The curve plot showing the $-\log 10$ p-values of all the 841 features across all the permutation scenarios. Each curve line represents the same microbiome feature. We can see that the $-\log 10$ p-values of the top hits generally remain on the top from the beginning to the end. For the full permutation in the end, all the $-\log 10$ p-values converge to the same median value which is close to 0.

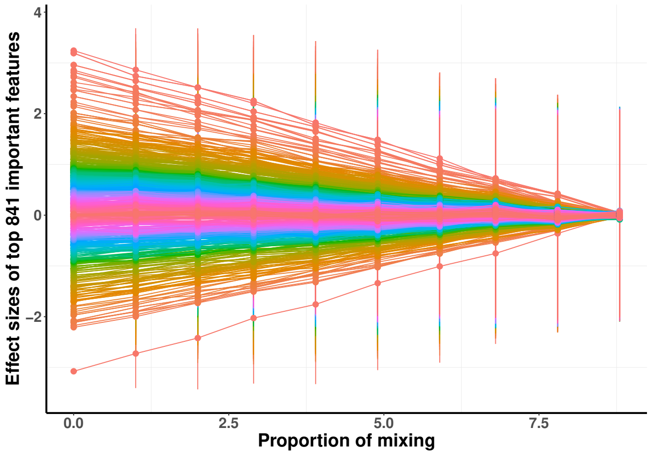

(c) The curve plot showing the effect sizes of all the 841 features across all the permutation scenarios. Each curve line represents the same microbiome feature. We can see that the absolute effect sizes of the top hits generally remain bigger from the beginning to the end. For the full permutation in the end, all the effect sizes converge to the same median value which is close to 0.

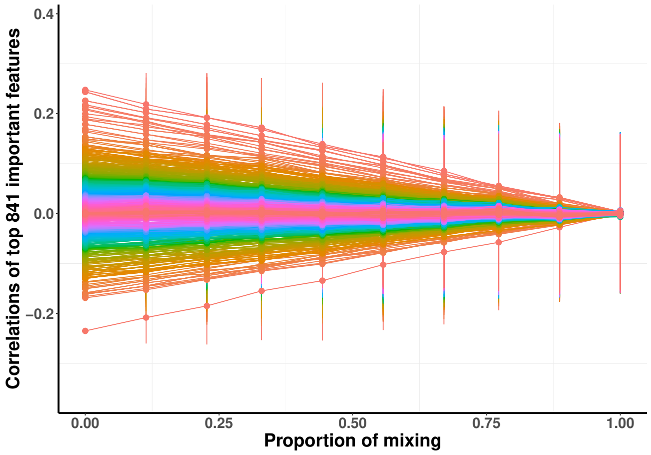

(d) The curve plot showing the correlations of all the 841 features across all the permutation scenarios. Each curve line represents the same microbiome feature. We can see that the absolute correlation of the top hits generally remain bigger from the beginning to the end. For the full permutation in the end, all the correlations converge to the same median value which is close to 0.

FIG. S11: The curve plots showing number of significant features, ordered $-\log 10$ p-values, ordered effect sizes and ordered correlations across permutation scenarios.

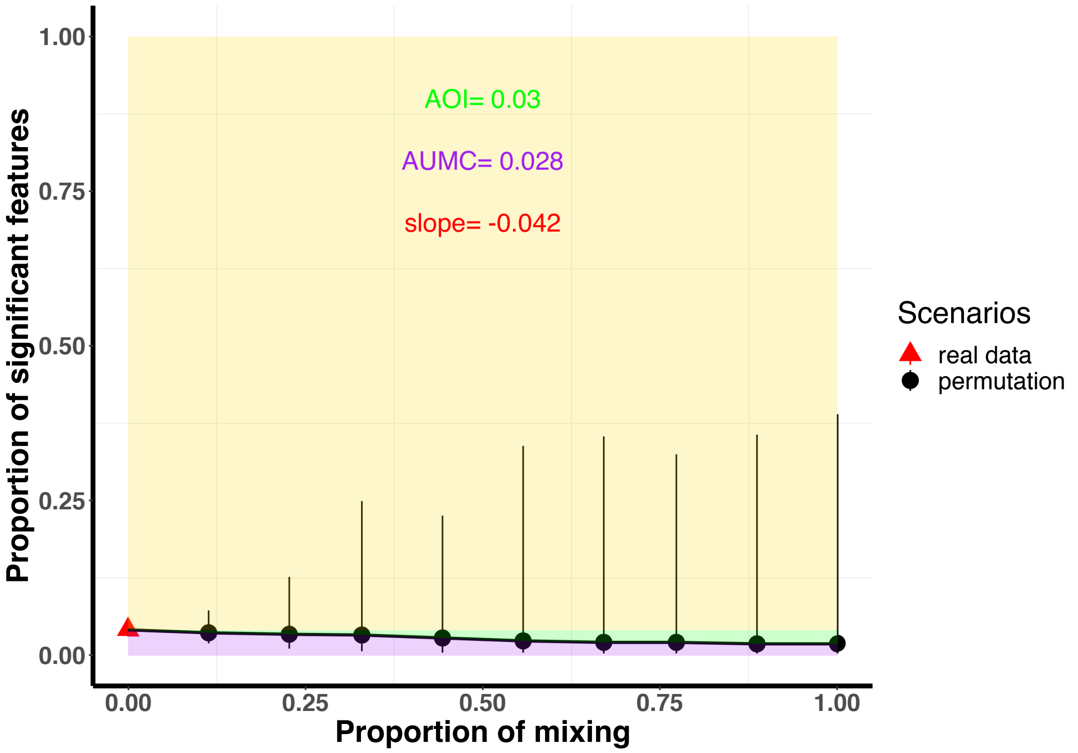

FIG. S12: The curve plot showing the proportion of significant features across permutation scenarios. The red triangle denotes the proportion of significant features in real data. The black point denotes the median of proportion of significant features in the permuted data. The vertical line denotes the 2.5%-97.5% quantile intervals of proportion of significant features in the permuted data. AOI is short for area of interest, which denotes the proportion of significant features out of all the features (area of green plus purple). AUMC is short for area under the mixing curve (area of purple). Slope denotes the slope of the red triangle.

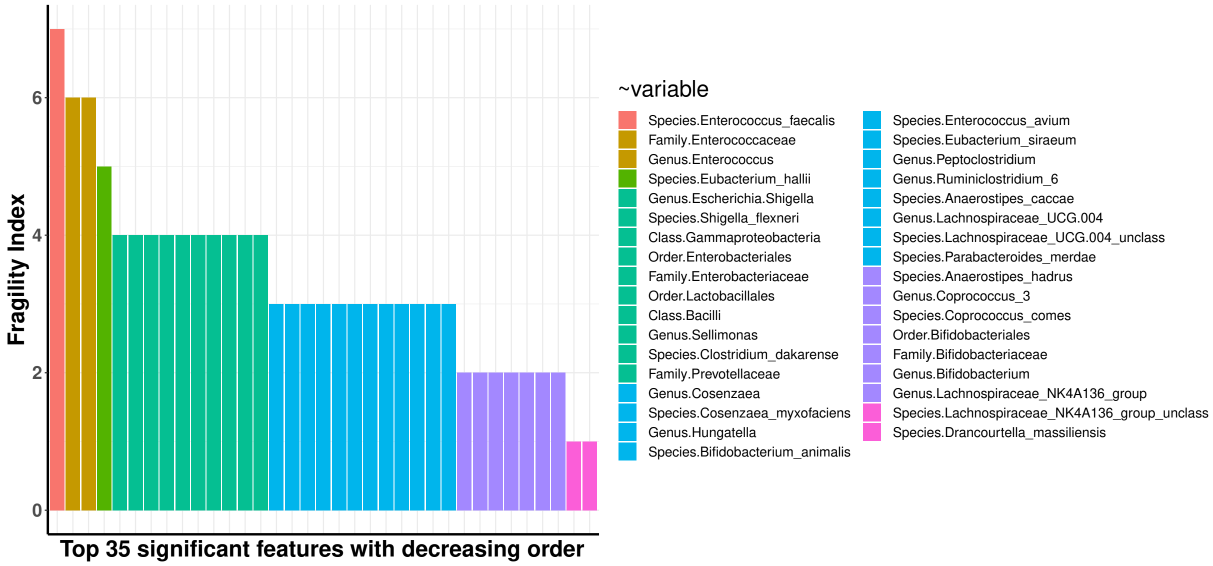

FIG. S13: Bar plot showing the fragility indices of the top 50 significant features with decreasing order. Each bar denotes a microbiome feature. The same color denotes microbiome features have the same fragility index.

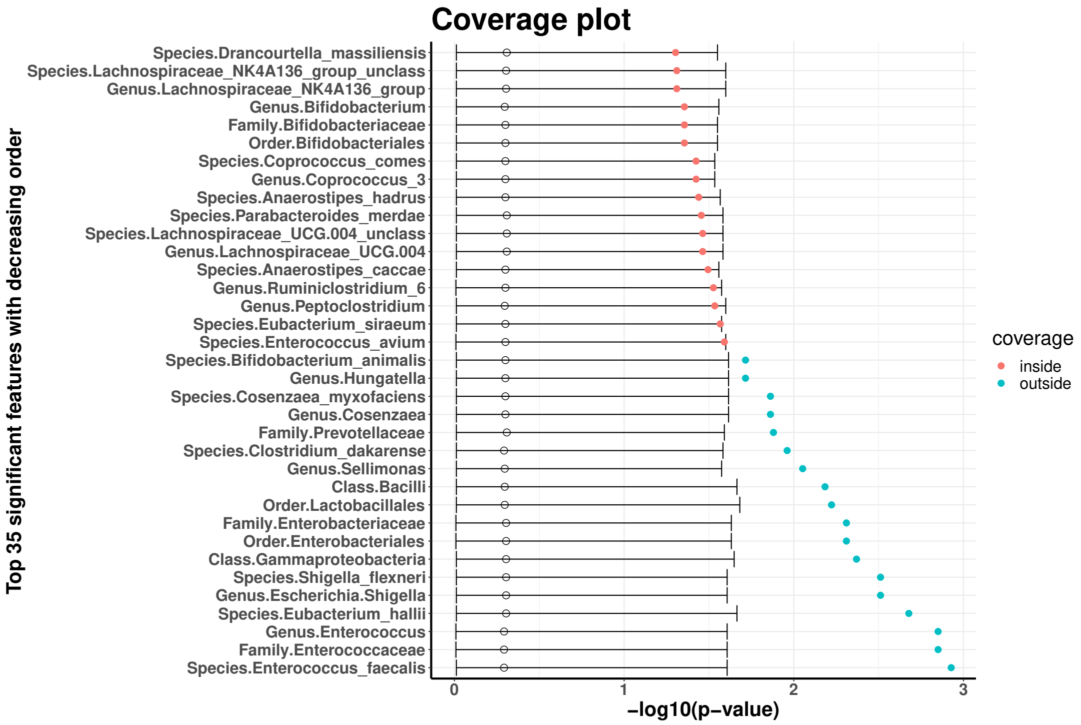

FIG. S14: Coverage plot of the top 50 features with decreasing order. The color dots denote the -log10(p-value) of top 50 features in the observed data (mixing proportion is 0). The horizontal bars describe the 95% quantile confidence intervals of the -log10(p-value) in the full permutation scenario (the mixing proportion is 1).

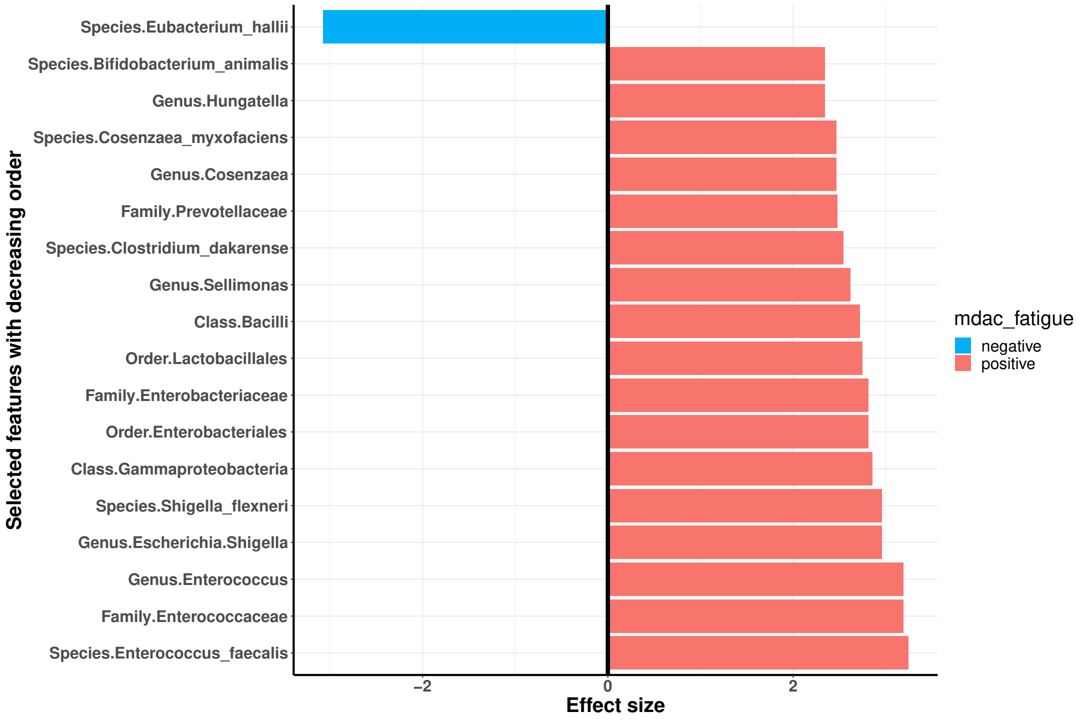

FIG. S15: Plot of effect sizes of identified features. The red bar denotes the positive effect size (microbiome features are associated with high fatigue). The blue bar denotes the negative effect size (microbiome features are associated with low fatigue).

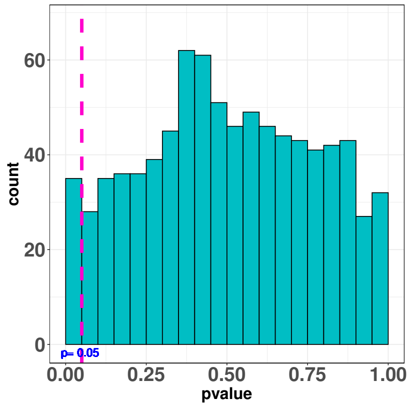

(a) Permutation proportion is 0

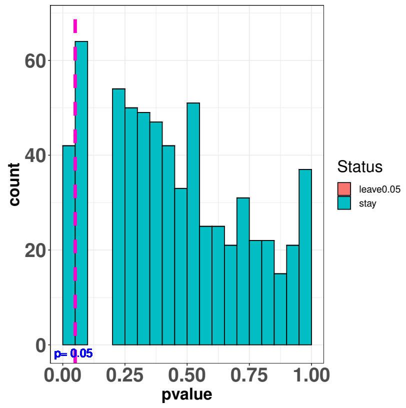

(b) Permutation proportion is 0.11

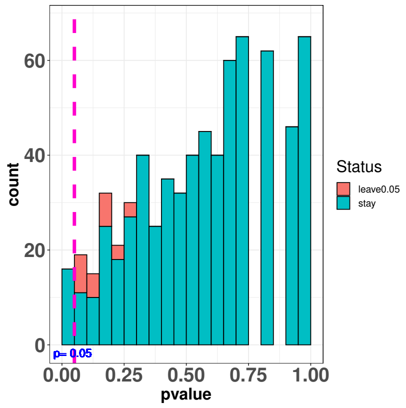

(c) Permutation proportion is 0.22

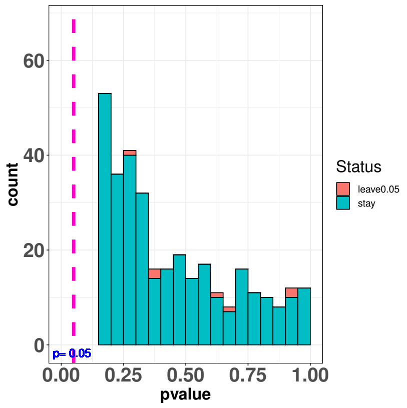

(d) Permutation proportion is 0.33

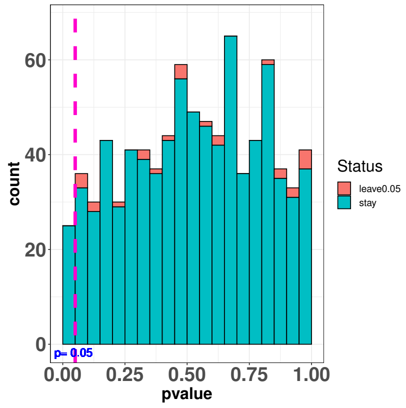

(e) Permutation proportion is 0.44

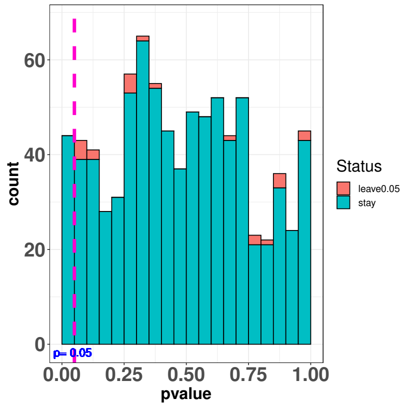

(f) Permutation proportion is 0.56

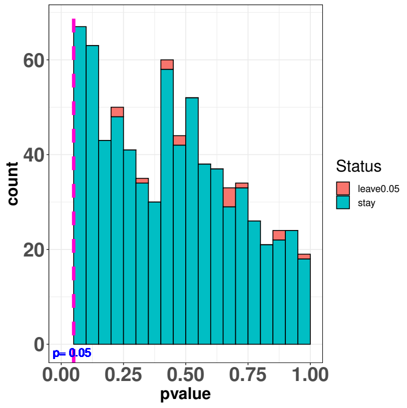

(g) Permutation proportion is 0.67

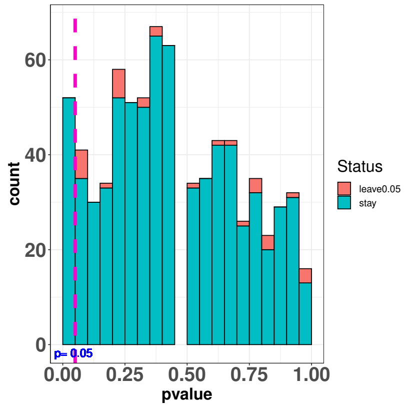

(h) Permutation proportion is 0.78

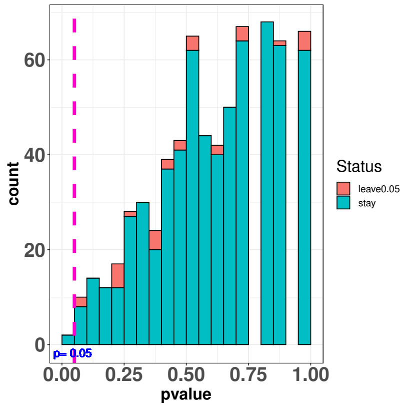

(i) Permutation proportion is 0.89

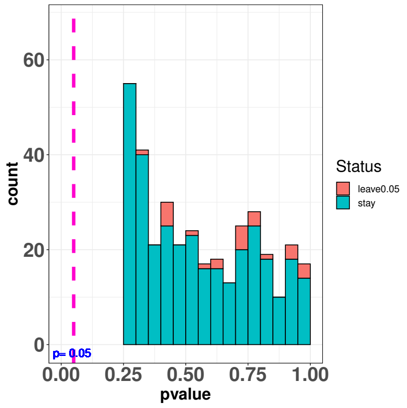

(j) Permutation proportion is 1

FIG. S16: The distribution of p-values across permutation scenarios. The x-axis represents the p-values. The width of each bar is 0.05, so there are 20 bars in each plot. The y-axis represents the frequency of p-values in each category. The red color marks the number of significant p-values (less than 0.05) transiting to nonsignificant categories (more than 0.05).
